# Copper-induced injectable hydrogel with nitric oxide for enhanced immunotherapy by amplifying immunogenic cell death and regulating cancer associated fibroblasts

**DOI:** 10.1186/s40824-023-00389-4

**Published:** 2023-05-10

**Authors:** Shuilin Shen, Zimeng Zhang, Haixiao Huang, Jing Yang, Xinyue Tao, Zhengjie Meng, Hao Ren, Xueming Li

**Affiliations:** School of Pharmaceutical Science, Nanjing Tech University, Nanjing, 211816 Jiangsu China

**Keywords:** Injectable hydrogel, Nitric oxide, Immunotherapy, Immunogenic cell death, Cancer associated fibroblast

## Abstract

**Background:**

Immunogenic cell death (ICD) induced by different cancer treatments has been widely evaluated to recruit immune cells and trigger the specific antitumor immunity. However, cancer associated fibroblasts (CAFs) can hinder the invasion of immune cells and polarize the recruited monocytes to M2-type macrophages, which greatly restrict the efficacy of immunotherapy (IT).

**Methods:**

In this study, an injectable hydrogel induced by copper (Cu) has been designed to contain antibody of PD-L1 and nitric oxide (NO) donor. The therapeutic efficacy of hydrogel was studied in 4T1 cells and CAFs in vitro and 4T1 tumor-bearing mice in vivo. The immune effects on cytotoxic T lymphocytes, dendritic cells (DCs) and macrophages were analyzed by flow cytometry. Enzyme-linked immunosorbent assay, immunofluorescence and transcriptome analyses were also performed to evaluate the underlying mechanism.

**Results:**

Due to the absorbance of Cu with the near-infrared laser irradiation, the injectable hydrogel exhibits persistent photothermal effect to kill cancer cells. In addition, the Cu of hydrogel shows the Fenton-like reaction to produce reactive oxygen species as chemodynamic therapy, thereby enhancing cancer treatment and amplifying ICD. More interestingly, we have found that the released NO can significantly increase depletion of CAFs and reduce the proportion of M2-type macrophages in vitro. Furthermore, due to the amplify of ICD, injectable hydrogel can effectively increase the infiltration of immune cells and reverse the immunosuppressive tumor microenvironment (TME) by regulating CAFs to enhance the therapeutic efficacy of anti-PD-L1 in vivo.

**Conclusions:**

The ion induced self-assembled hydrogel with NO could enhance immunotherapy via amplifying ICD and regulating CAFs. It provides a novel strategy to provoke a robust antitumor immune response for clinical cancer immunotherapy.

**Graphical Abstract:**

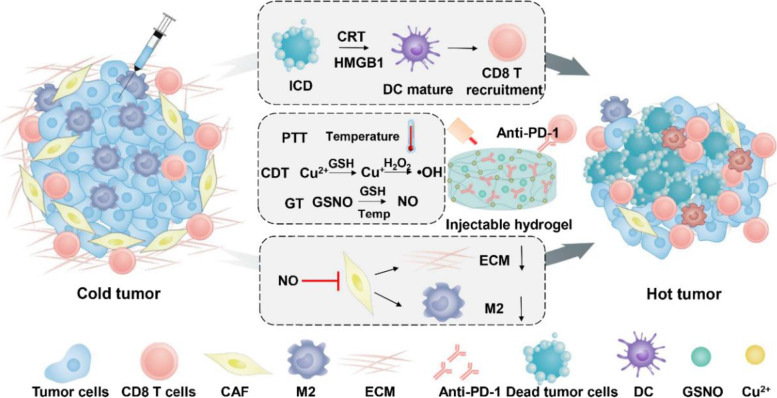

**Supplementary Information:**

The online version contains supplementary material available at 10.1186/s40824-023-00389-4.

## Introduction

Immunotherapy (IT), in particular immune checkpoint blockade (ICB) has attracted tremendous attention in clinical cancer therapy through stimulating the anti-tumor immune system to eradicate tumor cells. Compared to traditional treatments, it can effectively prevent tumor recurrence and metastasis to prolong the survive of patients. However, due to the low immunogenicity of cancer cells and insufficient tumor-infiltrating cytotoxic T cells (CTLs), the efficacy of ICB based on cancer immunotherapy showed low response rate in clinical application [1]. In addition, some of the infiltration immune cells in the tumor are mostly transformed into tumor-promoting immunosuppressive cells, which further restricted the therapeutic efficacy [2, 3]. Previous studies have been proved that the tumor cells undergoing immunogenic cell death (ICD) release tumor-associated antigens to activate anti-tumor immune effect and recruit CTLs [4–6]. Some traditional therapies, like radiation therapy (RT), chemotherapy (CT) and photodynamic therapy (PDT) are effective ways to induce ICD, and they were reported to achieve better efficacy when combined with ICB [7–9]. Photothermal therapy (PTT) is also an efficient strategy to trigger ICD cascade with hyperthermia for local cancer treatment and elicit an anti-tumor immune response by promoting the infiltration of CTLs [10–12]. Moreover, several studies including ours have demonstrated that PTT combined with ICB immunotherapy can significantly enhance the therapeutic efficacy of cancer and reduce tumor metastasis [13–16]. Therefore, the combination therapy of ICB and other treatments is an effective way to realize immunologically “cold” tumor to “hot”, and thus strengthening cancer immunotherapy. However, researchers have also found that during the treatment, the immunosuppressive tumor microenvironment (TME) can severely hinder the infiltration of immune cells and weaken the anti-tumor immune effect in some extent, which reduce the therapeutic efficacy.

Cancer associated fibroblasts (CAFs) is one of the most abundant stromal cells in TME to secret a variety of tumor-promoting growth factors and provide great convenience for tumor proliferation, migration and invasion [17–19]. Furthermore, it is the huge driving force to cause immunosuppression. By shaping the external matrix of tumor, it can form a permeation barrier to prevent the deep infiltration of drugs and immune CTL cells into tumor tissues, resulting in immunologically “cold” tumors [20]. Besides, a variety of cytokines secreted by CAFs, such as matrix metalloproteinases (MMPs), interleukin-6 (IL-6) and transforming growth factor-β (TGF-β), can promote immune cells to “serve” tumor, especially tumor associated macrophages (TAMs), resulting in immunosuppressive TME for tumor development [21, 22]. It has been reported that pro-tumor M2 phenotype TAMs dominate absolutely in the TME and showed a major limiting factor in the effectiveness of cancer immunotherapy [23, 24]. As the most prominent immune cells near the CAFs dense region, the M2 phenotype TAMs have been confirmed to be transformed from monocytes by TGF-β and other cytokines secreted by CAFs [25–27]. All these leave ICB unable to deeply penetrate into the tumor and reduce the external assistance of T cells and other immune cells [28–30]. Therefore, the treatment of CAFs is of great significance to promote the infiltration of immune cells and ICB drugs and reverse the immunosuppressive TME of TAMs.

As an immunomodulatory small molecule, nitric oxide (NO) can be produced in a variety of cells, and plays a very important role in killing invading bacteria, fungi and other microorganisms, tumor cells and inflammatory injury [31, 32]. At present, many studies have shown that high dose of NO (> 500 nM) is tumoricidal and can be used alone or in conjunction with other treatment modalities [33]. Zhang et al. designed an implantable NO release device, which controlled the release of NO through wireless charging irradiation and had a significant inhibitory effect on the *in-situ* tumor growth [34]. In previous studies, NO has been widely reported to improve the anti-tumor effect by regulating the normalization of tumor vascular function, enhancing tumor permeability and accelerating the accumulation of drugs and CTLs in tumors [35, 36]. This may be due to that the generation of NO in tumor site can promote the degradation of the intrinsic extracellular matrix (ECM) by activating MMPs to break collagen fibers. In addition, we have found that NO can block tumor-specific platelet function to increase the accumulation of drug and enhance T cell infiltration, thus inhibiting tumor metastasis [37]. Moreover, we also found that it can expand and activate dendritic cells (DCs) and reduce M2 macrophages, which enhances immunoreaction [35, 38]. As described above, the CAF showed significance in the remodeling of ECM and regulation of M2 macrophages for immunosuppressive TME. Therefore, it is urgently to realize the link between CAFs and NO treatment in regulation of immunosuppressive TME and apply NO for enhancement of immunotherapy.

**Fig. 1:**
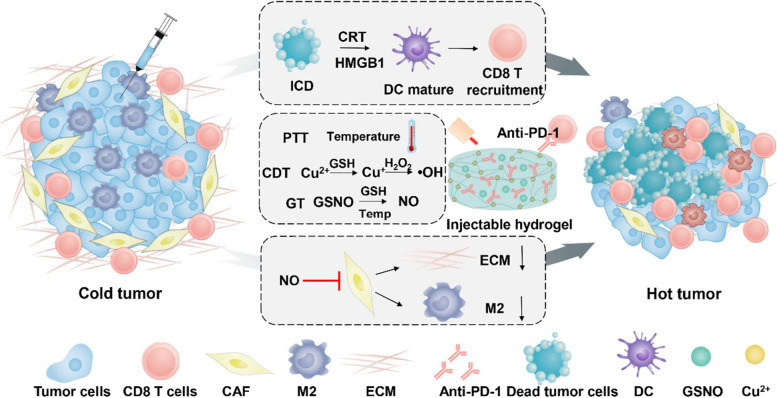
The scheme for the copper-induced injectable hydrogel and its pathway in enhanced cancer immunotherapy via amplified ICD and depletion of CAFs. The synergistic therapy of formed hydrogel amplified ICD to mature the DC and increase the recruitment of CD8 T cells. Furthermore, the NO released from formed hydrogel could interfere with CAFs to decrease the ECM and differentiation M2 macrophages to reverse immunosuppressive TME. With the antibody of PD-1, the self-assembled hydrogel could enhance the immunotherapy with enhanced ICD and depletion of CAFs

Previous studies including us have developed different kinds of nanoparticles to deliver NO for cancer treatment. However, due to the complex composition of body fluids, NO donor is easily affected by external factors such as metal, pH and temperature, which may have the disadvantages of instability, toxicity, short retention time and low bioavailability [39]. The injectable, *in-situ*-forming hydrogels have been widely used to control and sustain release of different bioactive agents, due to their low systemic toxicity and high treatment efficacy. Previous studies have also confirmed that the hydrogel could control release of NO or drugs in the targeted tumor site without systemic toxicity. In this study, we designed an injectable hydrogel based on the chelation of mercaptohyaluronic acid and copper ions (Cu^2+^) to encapsulate antibody of PD-L1 and NO donor (S-Nitrosoglutathione, GSNO), as shown in Fig. 1. Compared with traditional hydrogels, due to the photothermal conversion performance of Cu, the hydrogel showed hyperthermia as PTT to eradicate cancer cells for local cancer treatment with NIR laser irradiation [40]. In addition, the Cu^2+^ from injectable hydrogel can be reduced to Cu^+^ by high expression of glutathione (GSH) under the action of tumor acidic environment, which further reacted with hydrogen peroxide (H_2_O_2_) to produce cytotoxic hydroxyl radical (·OH) for depletion of cancer cells [41]. The increased temperature and high concentration of GSH could also increase NO generation from GSNO to kill cancer cells. These combination therapy could significantly amplify ICD of tumor cells, which could increase the maturation of DCs and in turn activate T cells and other immune cells. More interestingly, we have found that the activated CAFs are more sensitive for NO gas, rather than CDT and PTT. The death of CAFs reduce the secretion of TGF-β, leading to the recruited monocytes at tumor site not to differentiate into M2-type macrophages, which reverse the immunosuppressive TME. In addition, the ECM was degraded with the depletion of CAFs, which increase the infiltration of CTLs. With the loss of Cu^2+^, the hydrogel was degraded and release anti PD-L1 to increase the immunotherapy of recruited CTLs. This work illustrates the promising strategy to enhance immunotherapy of ICB with ion induced self-assembled hydrogel and reveal the NO regulation of CAFs in the immunosuppressive TME.

## Materials and methods

### Materials

Hyaluronic acid (HA, 90 ~ 100 kDa) was purchased from Shyuanye (Shanghai, China). EDC·HCl and CuCl_2_ were supported by Aladdin (Shanghai, China). NHS was obtained from Bidepharm (Shanghai, China). Rabbit anti-α-SMA, rabbit anti-FAP-α and donkey anti-rabbit IgG-AlexaFlour 488 were purchased from Absin (Shanghai, China). Neocuproine hydrochloride monohydrate was obtained from Macklin (Shanghai, China). Griess Kit, NO fluorescent probe and BCA protein assay kit were purchased from Beyotime (Shanghai, China). Cell viability (live dead cell staining) detection kit and Reactive Oxygen Species Assay kit were obtained from Keygen (Jiangsu, China). Mouse TGF-β Elisa kit and ATP Microplate Assay Kit was supported by Absin (Shanghai, China). All the antibodies were purchased from Biolegend (San Diego, CA, USA). HMGB1 Elisa kit was supported by Tongwei (Shanghai, China). Anti PD-L1 antiboby was purchased from Bio X Cell (West Lebanon, New Hampshire, USA).

### Preparation of the hydrogel

HA-SH were synthesized according to previous reports [42]. HA (0.5 g, 90 ~ 100 kDa) were dissolved in 50 mL of distilled water, and then, EDC·HCl (0.958 g) and NHS (0.575 g) were added to activate the carboxyl groups of HA. The pH of the reaction mixture was adjusted to 4.5 by HCl (0.5 mM). Then cystamine dihydrochloride (0.843 g) was added to introduce thiol groups. After 48 h reaction, the pH of the mixture was adjusted to 7.5 by NaOH (0.5 mM). Subsequently, DTT (2.313 g) was added to reduce disulfide bonds. The pH of the mixture was finally adjusted to 5 by HCl (0.5 mM) 24 h later. The reaction solution was first dialyzed (cut-off, Mw 3500 Da) against dilute HCl (pH = 5) containing 4 g·L^−1^ NaCl and followed by dialysis against dilute HCl (pH = 5). The purified solution was lyophilized and stored at -20 ℃. In order to investigate whether HA is introduced by sulfhydryl group, the infrared detection of lyophilized HA-SH was carried out. To make a comparison, HA alone was also tested by infrared detection.

To obtain an injectable hydrogel, the synthesized HA-SH (10 mg·mL^−1^) was dissolved and mixed with different concentrations of CuCl_2_ solution. Through chelating sulfhydryl groups with copper ions, a hydrogel with a three-dimensional network structure was constructed.

### Rheological test

A dynamic shear rheometer was used to conduct this Rheology experiments. Before the measurement, a cylindrical hydrogel with a diameter of 20 mm and a thickness of 1.5 mm was prepared and placed on the middle of a 20 mm diameter parallel plate with a proper gap. The time-sweep of the hydrogel was carried out at 37 °C, a frequency of 1 Hz and a strain of 1%.

### Photothermal effect of Cu-induced hydrogel

The synthesized HA-SH was dissolved in distilled water to form a solution at concentration of 20 mg·mL^−1^. Then, 200 µL of HA-SH solution was mixed with 200 µL of CuCl_2_ solution in 96-well plate to form hydrogel. 200 µL of HA-SH and CuCl_2_ solution were respectively mixed with 200 µL of distilled water as comparison groups. An 808 nm laser at a power density of 0.8 W·cm^−2^ was used to irradiate each group for 140 s. The temperature of each samples was measured and recorded by a K-type thermometer. To evaluate the photothermal stability of formed hydrogel, 400 µL of hydrogel was placed in 96-well plate and irradiated for five cycles with an 808 nm laser on and off at a power density of 0.8 W·cm^−2^.

In vivo, hydrogel (CuCl_2_, 0.92 mg·mL^−1^, HA, 10 mg·mL^−1^)was injected into the tumor site in the 4T1 tumor-bearing mice and irradiated with an 808 nm laser. The changes of the temperature were monitored by an infrared thermal camera.

### Hydrogel degradation behaviors

Two identical hydrogels with a volume of 2 cm^3^ were placed in vials and respectively covered with 1 mL of GSH (10 mM) solution and HCl (pH = 5) solution. The degradation states of hydrogels after 24 h were observed and photographed.

### Reduction of Cu^2+^ to Cu^+^

To confirm that Cu^+^ can be obtained by reducing Cu^2+^ through GSH, neocuproine hydrochloride monohydrate was applied to detect the generation of Cu^+^. CuCl_2_ solution with or without GSH was added to ethanol solution of neocuproine hydrochloride monohydrate. After incubation, the samples were scanned by UV–vis absorption spectroscopy at 360 ~ 600 nm.

### Generation of ROS

Two typical colorimetric method based on Methylene blue (MB) and 3,3′,5,5′-tetramethylbenzidine (TMB) were carried out to detect the ROS production by the Fenton-like reaction of Cu. Acetate buffer at pH 5.4 was used as solvent to simulate the tumor micro-acidic environment. Cu-based Hydrogels with different concentrations of CuCl_2_ (0.3, 0.5 and 0.8 mM) along with H_2_O_2_ (10 mM) and GSH (2.5 mM) were added to MB (5 µg·mL^−1^) and incubated on a shaker at 37 °C. After incubation with different time, the samples were scanned by UV–vis absorption spectroscopy at 580 ~ 700 nm.

In addition, 0.2 mg·mL^−1^ of TMB mixed with hydrogel (0.4 mM CuCl_2_) and varied concentration of H_2_O_2_ (5, 10, 20 mM) was incubated in tubes and placed on a shaker at 37 °C. At the desired interval time, all the groups were measured at 650 nm by a microplate reader.

### NO release

The GSNO was synthesized according to our previous studies [43]. To evaluate the release of NO from G@Gel, GSNO (0.2 mg·mL^−1^, 1.95%) was encased in hydrogel and added in 96-well plate mixed with GSH (10 mM). The released NO was measured by Griess Kit (Beyotime). Furthermore, hydrogel with GSNO was irradiated for different time to investigate the effect of NIR on the release of NO. The method was the same as described above.

### Protein release from the hydrogel

20 mg of HSA (200 mg·mL^−1^) was mixed with HA-SH solution first and then CuCl_2_ was added to form samples of hydrogel. The samples were divided into two groups and severally placed in 4 mL of buffer solution with pH 5.4 and pH 7.4. Besides, all the samples were incubated on a shaker at 37 °C. At the desired interval time, the media of the both groups were collected and determined with a BCA protein assay kit (Beyotime) according to the standard protocols to quantitate the amount of the released HSA.

### Cytotoxicity evaluation of 4T1 cells in vitro

The 4T1 cells were seeded in 96-well plates at a density of 1.0 × 10^5^ cell per well. After incubation overnight at 37 ℃, they were further co-cultured with hydrogel (Gel) and hydrogel containing GSNO (G@Gel) for 24 h. Each group was divided into with and without NIR light. The hydrogels with different concentrations of GSNO (0.05, 0.1, 0.15 and 0.3 mg·mL^−1^) and CuCl_2_ (0.1, 0.2, 0.3, 0.35 and 0.4 mM) were incubated with 4T1 cells for 24 h. Each well was carefully washed with PBS after various treatments. Then, 80 µL of RPMI Medium 1640 was added to each well along with 20 µL of 3-(4,5-dimethylthiazol-2-yl)-2,5-diphenyltetrazolium bromide (MTT, 5.0 mg·mL^−1^). The 96-well plates were incubated for an additional 4 h at 37 °C. At the end of the incubation, the supernatant fluid was diverted. To dissolve the resultant formazan crystals of the alive cells, 150 µL of DMSO was added to each well and measured at the wavelengths of 492 nm to calculate the cytotoxicity. In addition, the staining of live and dead cells was stained with PI (red fluorescence) and calcein AM (green fluorescence) by a kit (Keygen) and photographed under a fluorescence microscopy.

### Intracellular ROS detection

The 4T1 cells were seeded in 96-well plates at a density of 1.0 × 10^5^ cell per well and incubated overnight. Then, Gel and G@Gel were added for further incubation (CuCl_2_, 0.25 mM, 0.415%; GSNO, 0.2 mg·mL^−1^, 1.95%). Each treatment was divided into two groups (with or without light). After 24 h, intracellular ROS was detected by Reactive Oxygen Species Assay kit (Keygen) and the fluorescence images were captured by fluorescence microscopy.

### ATP and HMGB1detection in supernatant of 4T1 cells

The 4T1 cells were seeded in 96-well plates at a density of 1.0 × 10^5^ cell per well and incubated overnight. Then, Gel and G@Gel were added for further incubation (CuCl_2_, 0.25 mM, GSNO, 0.2 mg·mL^−1^). Each treatment was divided into two groups (with or without light). After 24 h, the ATP and HMGB1content in supernatant of 4T1 cells was evaluated by ATP Microplate Assay Kit and HMGB1 Elisa kits, according to the standard protocols.

### Transform of NIH 3T3 cells into CAFs via TGF-β

The NIH 3T3 cells were seeded in 6-well plates at a density of 1.0 × 10^6^ cell per well. After incubation overnight at 37 ℃, TGF-β was added and co-incubated for another 24 h to turn NIH 3T3 cells into CAFs (TGF-β, 10 ng·mL^−1^). Rabbit anti-α-SMA (Absin, abs130621), rabbit anti-FAP-α (Absin, abs149525) and donkey anti-rabbit IgG-AlexaFlour 488 (Absin, abs20020) were used to mark α-SMA and FAP-α. The up-regulation of α-SMA and FAP-α was then determined by fluorescence microscope.

### Cytotoxicity evaluation of CAFs in vitro

The TGF-β activated NIH 3T3 cells were seeded in 96-well plates at a density of 1.0 × 10^5^ cell per well and incubated overnight. Gel and G@Gel were then added separately and co-incubated for another 24 h (CuCl_2_, 0.25 mM, GSNO, 0.2 mg·mL^−1^). Each group was divided into groups with and without light. In the with light groups, each well was irradiated with an 808 nm laser for 1 min. After incubation for 24 h, the cell viability of CAFs was detected by CCK-8 according to the protocols.

### Inhibition of CAFs from secreting TGF-β

The TGF-β activated NIH 3T3 cells were seeded in 6-well plates at a density of 1.0 × 10^6^ cell per well and incubated overnight. Then, after carefully washed with PBS for twice, Gel and G@Gel were then added separately and co-incubated for another 24 h (CuCl_2_, 0.25 mM, GSNO, 0.2 mg·mL^−1^). Each group was divided into groups with and without light. Each well was irradiated with an 808 nm laser for 1 min. After incubation, the supernatant was removed and fresh medium was added for further incubation. After 48 h, the concentration of TGF-β in supernatant was detected by Elisa kit (Absin) according to the standard protocols.

### The uptake of NO by CAFs

The TGF-β activated NIH 3T3 cells were seeded in 6-well plates at a density of 1.0 × 10^6^ cell per well and incubated overnight. Gel and G@Gel were then added separately and co-incubated for another 24 h (CuCl_2_, 0.25 mM, GSNO, 0.2 mg·mL^−1^). The uptake of NO by CAFs was detected by a Kit (Beyotime) and monitored by fluorescence microscopy.

### The evaluation of M2 macrophages by inhibiting CAFs

The TGF-β activated NIH 3T3 cells were seeded in 6-well plates at a density of 1.0 × 10^6^ cell per well and incubated overnight. Gel and G@Gel were then added separately and co-incubated for another 24 h (CuCl_2_, 0.25 mM, GSNO, 0.2 mg·mL^−1^). Each group was divided into groups with or without NIR irradiation. After incubation for 24 h, the supernatant was removed and fresh medium was added for further incubation. After incubation for 48 h, the supernatant of TGF-β activated NIH 3T3 cells was then added into the RAW264.7 cells and co-incubated for 24 h. Anti-CD206-APC (Biolegend, Clone: C068C2, Catalog: 141,708) antibody were applied to stain the M2 macrophages and determined by flow cytometry.

### Transcriptome analyses of Gel- and G@Gel-treated CAFs

The TGF-β activated NIH 3T3 cells were seeded in 6-well plates at a density of 1.0 × 10^6^ cell per well and incubated overnight. Gel and G@Gel were then added separately and co-incubated for another 24 h (CuCl_2_, 0.25 mM, GSNO, 0.2 mg·mL^−1^). The transcriptome analyses were assisted by Majorbio. Measurements were taken from distinct samples (*n* = 3). The significant upregulation and downregulation of genes were mapped. Based on GO annotation, we also analyzed the changes of genes associated with biological process, cellular component, and molecular functions were analyzed. The KEGG pathway was analyzed to evaluate the function of related genes.

### The differentiation of RAW264.7 to M1-type macrophages induced by GSNO

The RAW264.7 cells were seeded in 6-well plates at a density of 1.0 × 10^6^ cell per well. After incubation overnight at 37 ℃, RAW264.7 were divided into control group and GSNO-treated group. In the GSNO-treated group, RAW264.7 cells were co-incubated with GSNO (GSNO, 0.2 mg·mL^−1^) for 24 h. After incubation, RAW264.7 cells were stained with anti-CD86-PE (Biolegend, Clone: GL-1, Catalog: 105,007) and detected by flow cytometry.

### Anti-tumor effect on 4T1 tumor-bearing mice

The anti-tumor effect of the injectable hydrogel was studied by 4T1 tumor-bearing mice. The 4T1 cells were uniformly suspended in RPMI Medium 1640 and subcutaneously injected into the right flank of each female BALB/c mouse. When tumor size reached about 200 mm^3^, they were randomly divided into seven groups (*n* = 5–7) and respectively injected with 100 µL of saline, free GSNO and aPD-L1 solution (G + aP), G@Gel, aP@Gel, G/aP@Gel (anti PD-L1, 1.0 mg·mL^−1^, 8.25%; GSNO, 0.2 mg·mL^−1^, 1.65%; CuCl_2_, 0.92 mg·mL^−1^, 7.59%). The mice in light irradiation group were irradiated with an 808 nm laser at a power density of 0.8 W/cm^2^ for 1 min after injection, which caused a mild photothermal effect at the tumor site. The tumor size and the body weight of the mice were then recorded. The calculation of tumor size is as the following formula: width^2^ × length × 0.5.All the tumors were removed from the mice on day 21 and photographed. Other major organs (i.e., heart, lung, liver, spleen and kidney) were collected on day 21 and carried out H&E to evaluate the toxicity of hydrogel. In addition, on day 5, the tumor after different treatments were also collected to performed Masson, H&E, TUNEL, Ki-67, SMA-α, Collagen1 and TGF-β staining according to standard protocols and the fluorescence images were captured by fluorescence microscope.

### Anti-tumor immune response of hydrogel in vivo

To evaluate the immune response after hydrogel treatment, tumors and lymph nodes were collected from the mice on fifth day for further analysis. They were digested with the prepared enzyme lysate and the cell suspension was obtained through the 70 µm sieve. The collected lymphocytes were incubated with anti-CD11c-FITC (Biolegend, Clone: N418, Catalog: 117,305), anti-CD80-APC (Biolegend, Clone: 16-10A1, Catalog: 104,714), and anti-CD86-PE (Biolegend, Clone: GL-1, Catalog: 105,007) antibodies according to the standard protocols, to determine the content of matured DCs in lymph nodes. The percentages of CTLs in tumor were determined by staining tumor cell suspension with anti-CD3-APC (Biolegend, Clone: 17-A2, Catalog: 100,235), and anti-CD8a-FITC (Biolegend, Clone: 53–6.7, Catalog: 100,706) antibodies according to the standard protocols. For evaluation of macrophages in tumor, they were stained with anti-CD11b-FITC (Biolegend, Clone: M1/70, Catalog: 101,206), anti-F4/80-PE/Cy7 (Biolegend, Clone: BM8, Catalog: 123,113), and anti-CD206-APC (Biolegend, Clone: C068C2, Catalog: 141,708) antibodies. The cell suspensions were determined and analysis by flow cytometry.

### Inhibition of distant tumor growth

To figure out the abscopal therapeutic effect in mice, the inhibition of distant tumor growth was conducted also by a 4T1 tumor model. First, 4T1 cells (3 × 10^6^) were injected into the right flank of each female BALB/c mouse to form primary tumors and the distant tumors were inoculated simultaneously with the primary tumors by injecting 4T1 cells (5 × 10^5^) into the left flank. Once, the volume of the primary tumor reached about 200 mm^3^, mice were randomly divided into five groups and the treatment was initiated as above (saline + L, G@Gel + L, aP@Gel + L, G/aP@Gel + L, G/aP@Gel-L, anti PD-L1, 1.0 mg·mL^−1^; GSNO, 0.2 mg·mL^−1^; CuCl_2_, 0.92 mg·mL^−1^). The volume of the primary and the distant tumor in mice were recorded to evaluate the abscopal therapeutic efficacy.

### Statistical analysis

All the data were given as mean ± standard deviation (SD) or the mean ± standard error of the mean (SEM) of at least three samples. One-way analysis of variance (ANOVA) and Tukey’s postdocs tests were used to make multiple comparisons. All the statistical analyses were conducted using GraphPad PRISM 5 software. The comparison of two groups was followed by unpaired Student’s t-test (two-tailed). The level of significance was defined as *p < 0.05, **p < 0.01.

## Results

### Synthesis and characterization of hydrogel

**Fig. 2:**
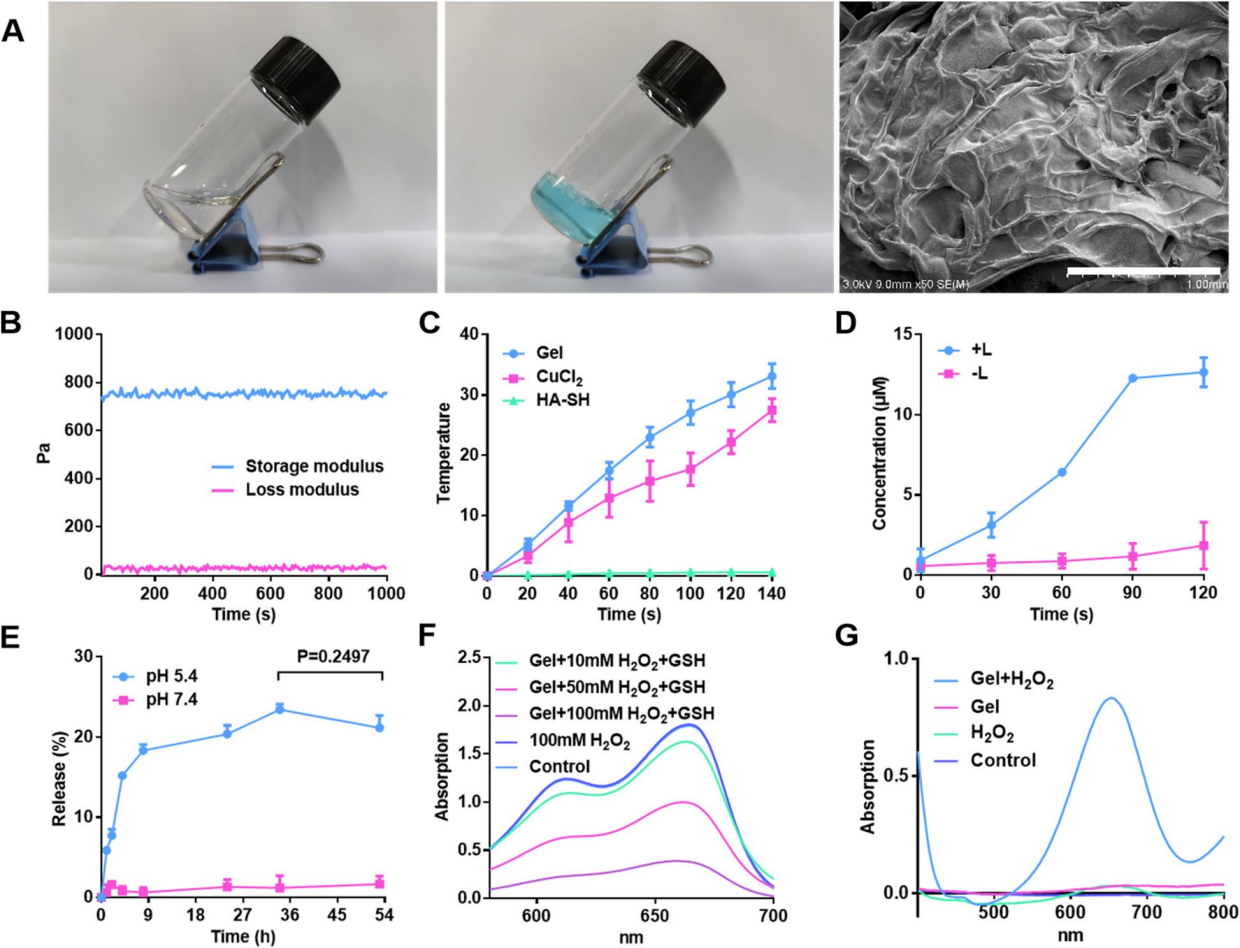
Characterization of the Cu-based hydrogel. **A** Photographs of the sol-to-gel transition and SEM image of the surface of the hydrogel. **B** Storage and loss modulus changes of the hydrogel at 37 °C, a frequency of 1 Hz and a strain of 1%. **C**.Temperature variation curve of hydrogel, CuCl_2_ solution and HA-SH solution with NIR laser irradiation. **D** Release profiles of NO from hydrogel with or without NIR laser irradiation. **E** Cumulative release profiles of HSA protein from hydrogel in different buffer solution (pH = 5.4 and 7.4). **F** MB degradation rate of Cu-based hydrogel after addition of GSH with different concentration of H_2_O_2_. **G** Generation of ·OH through the reaction of hydrogel and H_2_O_2_ by oxidation of TMB

Hyaluronic acid (HA), as a biocompatible macromolecule, has great advantages and is widely used to form anti-tumor immune hydrogels. As the chelation of Cu ions and sulfhydryl groups (-SH) to form hydrogel, HA was chemically modified with cystamine to introduce thiol groups according to previous studies [42]. As shown in Figure S1, compared with HA, HA with thiol groups (HA-SH) showed a weak sulfhydryl peak at 2300 cm^−1^ by infrared spectroscopy, which indicated thiol groups have been successfully incorporated to HA. As shown in Figure S2, the existence of thiol group in HA-SH reduced DTNB to TNB. All these results indicated the successful modification -SH to HA. As shown in Fig. 2A, lyophilized HA-SH dissolved in water exhibited as a transparent solution. Once mixed with Cu^2+^, ion-based hydrogels were formed in situ via S-Cu coordinative cross-linking. The surface structure of the hydrogel was confirmed by scanning electron microscope (SEM) and the interior structure indicated that the ion-induced hydrogel can firmly encase drugs for the application of cancer treatment (Figure S3). The storage modulus (G’) and the loss modulus (G’’) of the ion-induced hydrogel were measured to evaluate the mechanical properties. As shown in Fig. 2B, the rheological behaviors in the time sweep showed that the G’ and G’’ values of hydrogel remained the same with the time increased, indicating the formed hydrogel had stable performance and was not easily affected by external forces.

To further confirm the gelation status of the injected hydrogel in vivo, the hydrogel was injected subcutaneously into the mice and cut the skin, the gelation status of the injected hydrogel in vivo was then photographed. As shown in Figure S4, the hydrogel was formed in vivo once injected and did not degraded significantly after 7 days of injection, indicating the hydrogel had a stable capacity of retention in vivo and could be retained in body for at least 7 days.

### Photothermal effect of hydrogel in vitro

Copper-based nanoparticles have been reported to have photothermal conversion ability due to the occurrence of Cu ion [44]. Therefore, the photothermal conversion capacity of hydrogel in vitro was evaluated by recording the increased temperature. As shown in Fig. 2C, upon irradiated by NIR laser (808 nm, 0.8 W·cm^−2^), the temperature of CuCl_2_ solution and hydrogel increased about 28 °C and 35 °C within 140 s respectively, which is higher than that of HA-SH solution (approximately no change). The photothermal conversion efficiency of hydrogel and CuCl_2_ solution were 49.7% and 41.2%, respectively. These results suggested that the photothermal conversion capacity of hydrogel is caused by copper ions and was further confirmed that the successful chelation of Cu ions with thiol groups. Furthermore, the photothermal stability of hydrogel was evaluated as well. During five consecutive photothermal transformation, the hydrogel presented a good photothermal stability, which could reach the same temperature in about the same time (Figure S5). The hydrogel was then irradiated with an 808 nm laser and photographed before and after the irradiation. After irradiated for 1 and 3 min, the appearance and the flexibility of the hydrogel didn’t have any change, revealing an excellent stability of hydrogel (Figure S6). All these results indicated that the Cu-induced hydrogel could be used as a drug carrier with photothermal capacity for cancer treatment without any addition of photosensitizer.

### NO and drug release behavior of hydrogel in vitro

GSNO, a NO donor, was reported to release NO for cancer treatment at a rate affected by temperature, metal ions (e.g., Cu^+^) and light [45–47]. As shown in Figure S7, GSNO with or without GSNO showed little release of NO. While, The NO from G@Gel was released about 140 μM within 3 h after addition of GSH. This result indicated that G@Gel with GSH could significantly facilitate the release of NO due to the Cu^+^ reduction from Cu^2+^ by GSH. In order to confirm the idea, the neocuproine hydrochloride monohydrate was applied to detect the generation of Cu^+^, which could form a colored complex with neocuproine hydrochloride monohydrate, and has a maximum UV absorption at 460 nm. As shown in Figure S8, CuCl_2_ along with GSH presented a significantly larger absorption at 460 nm than CuCl_2_ alone, indicating the generation of Cu^+^ through the reduction of Cu^2+^ by GSH. The GSNO was sensitive to Cu^+^ for rapid release of NO. In addition, as shown in Fig. 2D, NO released much faster from hydrogel with NIR irradiation than without laser irradiation, approximately 6-folds. All these results indicated that the Cu-based hydrogel is suitable for GSNO encapsulation to enhance NO generation with GSH and NIR laser, which is better for cancer treatment. When without the irradiation of NIR, G@Gel rarely released NO even after 72 h, suggesting G@Gel could supply enough NO in a long term (Figure S9).

It is known to us that the tumor microenvironment showed high expression of GSH and acidic conditions [48, 49]. In addition, the chelation of sulfhydryl and copper ions has been reported to easily affect by the GSH and acidic conditions. Thus, the simulated degradation behavior of hydrogel in vitro was investigated with addition of GSH solution (10 mM) and HCl solution (pH = 5), as shown in Figure S10. The hydrogel was observed to be largely degraded after 24 h. These indicate that Cu-based hydrogel could effectively degrade under the appearance of GSH and acidic conditions for efficiently release of drugs at tumor site. In order to investigate whether anti PD-L1 could effectively release from hydrogel, the release behavior of protein in hydrogel was evaluated instead HSA of anti PD-L1 (Fig. 2E). As expected, nearly 20% of HSA was released from hydrogel after 8 h in pH 5.4 dissolution medium, while it was rarely released in pH 7.4 dissolution medium even after 53 h. This confirmed that our hydrogel degrades more easily under acidic conditions, thus anti PD-L1 can sufficiently release from hydrogel and exert anti-tumor immunotherapy.

### Cu-based hydrogel induced Fenton-like effect

It has reported that Cu^2+^ could be reduced to Cu^+^ by GSH, and then reacted with H_2_O_2_ to generate toxic hydroxyl radicals (·OH) via a Fenton-like reaction [50]. As proved above, the Cu^2+^ in formed hydrogel could be reduced to Cu^+^ by GSH. According to previous studies, the ·OH generation of Cu-based hydrogel was evaluated by degradation of MB and oxidized of TMB. As shown in Fig. 2F, with addition of GSH and H_2_O_2_ (100 mM) for 4 h, the absorption of 660 nm was decreased from 1.8 to 0.3, which indicated the generation of ·OH degrade MB. In addition, the hydrogels added with H_2_O_2_ (10 mM and 50 mM) still have more than 1.0 absorption. These results indicated that the ·OH generation was consistent with the concentration of H_2_O_2_, which react with Cu^+^. Furthermore, degradation of MB under different concentration of Cu^2+^ were also evaluated after incubation for 4 h and 19 h (Figure S11). This result showed that the generation of ·OH was increased with the concentration of Cu^2+^ in formed gels. Also, the generation of ·OH could oxidize TMB as blue, which showed a significant absorption at 650 nm. As shown in Fig. 2G, the generation of ·OH could increase the absorption of oxidized of TMB at 650 nm. In addition, the oxidation results of TMB was also enhanced with increased concentration of H_2_O_2_ at different time, as shown in Figure S12. All these results revealed that Cu-based hydrogel could generate ·OH as Fenton-like effect under high expression of GSH and H_2_O_2_, and further for cancer treatment.

### Cell viability of G@Gel in 4T1 cells

**Fig. 3:**
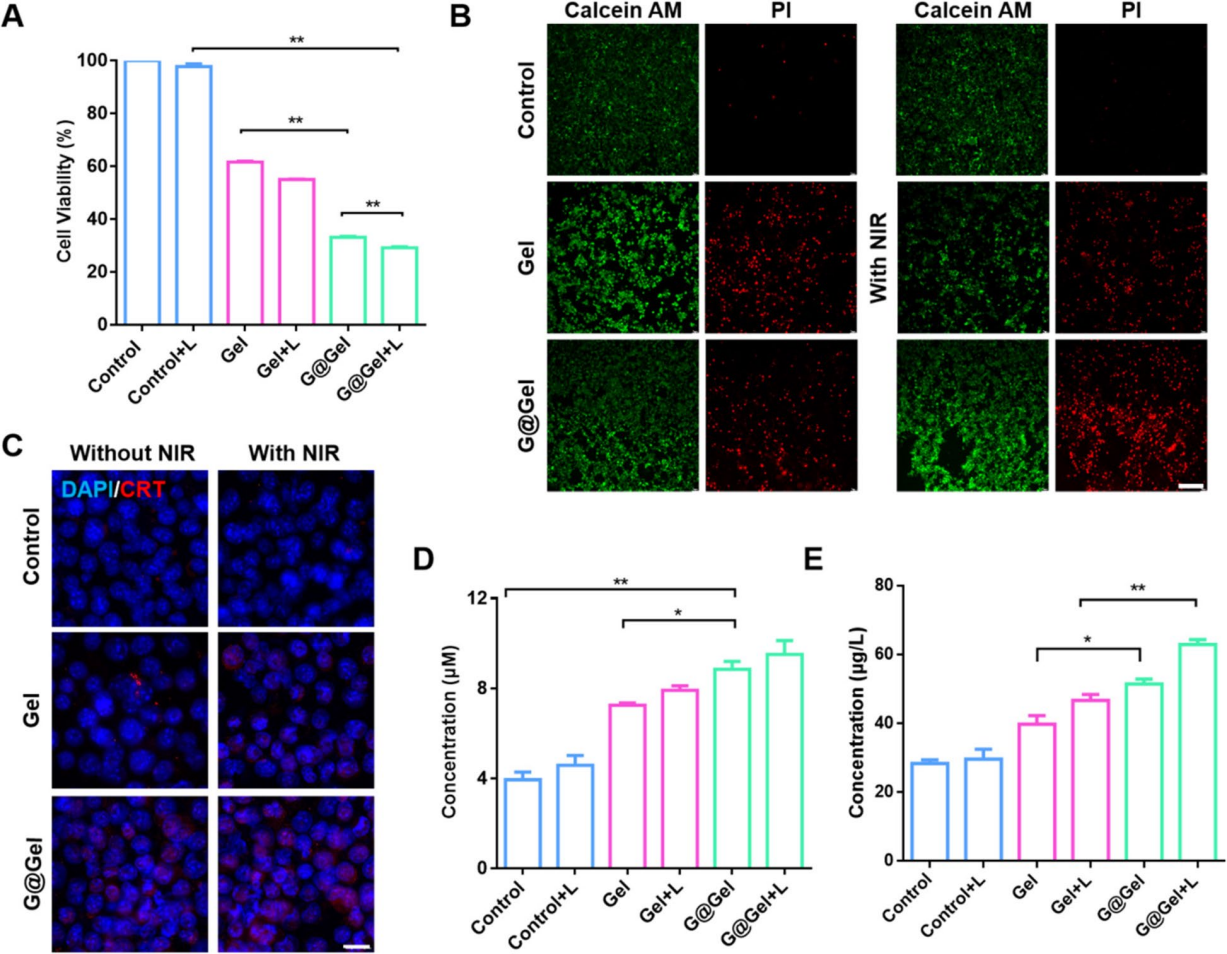
Cytotoxicity on 4T1 cells with the treatment of Gel and G@Gel with or without NIR in vitro. **A** Cell viability of 4T1 cells with different treatments. **B** Live and dead staining of 4T1 cells with various treatments (Scale bar = 200 µm). **C** Immunofluorescence staining of CRT in 4T1 cells (Scale bar = 20 µm). DAPI and CRT was stained blue and red, respectively. **D** ATP and(**E**) HMGB1 content in supernatant of 4T1 cells after various treatment. Data are shown as mean ± SD (*n* = 3). The comparison of two groups was followed by unpaired Student’s t-test (two-tailed). The level of significance was defined as *p < 0.05, **p < 0.01

As proved above, the formed Cu-based hydrogel could generate ·OH as CDT and induce hyperthermia as PTT in vitro. Therefore, the cytotoxicity of hydrogel in 4T1 was investigated through the MTT method. As shown in Fig. 3A, due to the occurrence of Fenton-like reaction, Cu-based Gel alone had a cytotoxic effect on 4T1 cells, in which the cell viability dropped to 60% after treatment. At the same time, the cell viability of G@Gel-treated group was 30%, only half that of Gel-treated group. This may be due to the generation of NO from GSNO catalyzed by Cu^+^ induce tumor cell apoptosis, which showed the combination therapeutic efficacy of CDT and NO gas [51]. To figure out the generation of ·OH in 4T1 cells, DCFH-DA as indicator of ROS has been applied to determine intracellular ROS. The Gel-treated and G@Gel-treated groups produced significantly more ROS than the control group, indicating a Fenton-like reaction induced by Cu-based hydrogel occurred in 4T1 cells (Figure S13). Meanwhile, compared with Gel-treated group, G@Gel-treated group generated more ROS, because NO can be oxidized to radical and nitro compounds with high oxidative activity.

Besides, there is no significant decline in the control group after irradiation, due to its lack of photothermal conversion capacity. In the group treated with Gel and G@Gel after irradiation, the cell viability was decreased than that without laser irradiation, because the Gel increased the temperature to kill cancer cells with PTT. All these results indicated that our formed G@Gel could inhibit tumor cell growth by the combination therapy of PTT, CDT and NO gas-based gas therapy (GT). In addition, as shown in Figure S14, cell viability decreased with increasing concentration of Cu^2+^ and GSNO, which indicated that the cytotoxicity of different groups was obviously dose-dependent. Furthermore, the fluorescence images of live and dead cells in each treatment were shown in Fig. 3B, which was consistent with the results of cell viability.

ICD of tumor cells was reported to produce tumor associated antigens, which can activate the maturation of DCs [52]. In order to evaluate the induction of ICD of tumor cells by Cu-based hydrogel, classical damage-associated molecular patterns (DAMPs), including CRT, HMGB1 and the release of ATP were investigated in 4T1 cells. As shown in Fig. 3C, there was no red fluorescence observed in the control group with CRT staining. After treatment of Gel and G@Gel, CRT was transferred to the cell membrane in a certain extent which could be stained by fluorescence probe. The fluorescence of CRT in 4T1 cells treated with Gel and G@Gel was brighter than that of control group. In addition, the relative percentages of CRT are shown in Figure S15. The results indicated that the CRT level in G@Gel with NIR is about 1.5-fold and 13-fold relative to Gel with NIR and control groups, respectively. Besides, the results of ATP and HMGB1 detection in supernatant of 4T1 cells are shown in Fig. 3D and E. The concentration of ATP in G@Gel-treated group increased more than onefold compared with control group, and is 1.2-fold relative to Gel group. The 4T1 cells treated with G@Gel plus NIR laser showed the most expression of CRT and HMGB1 and the release of ATP. All these results indicated that our formed G@Gel can effectively amplify ICD of 4T1 cells, which could further stimulate the maturation of DCs and induce anti-tumor immune response.

### Cell viability of CAFs by hydrogel in vitro

**Fig. 4:**
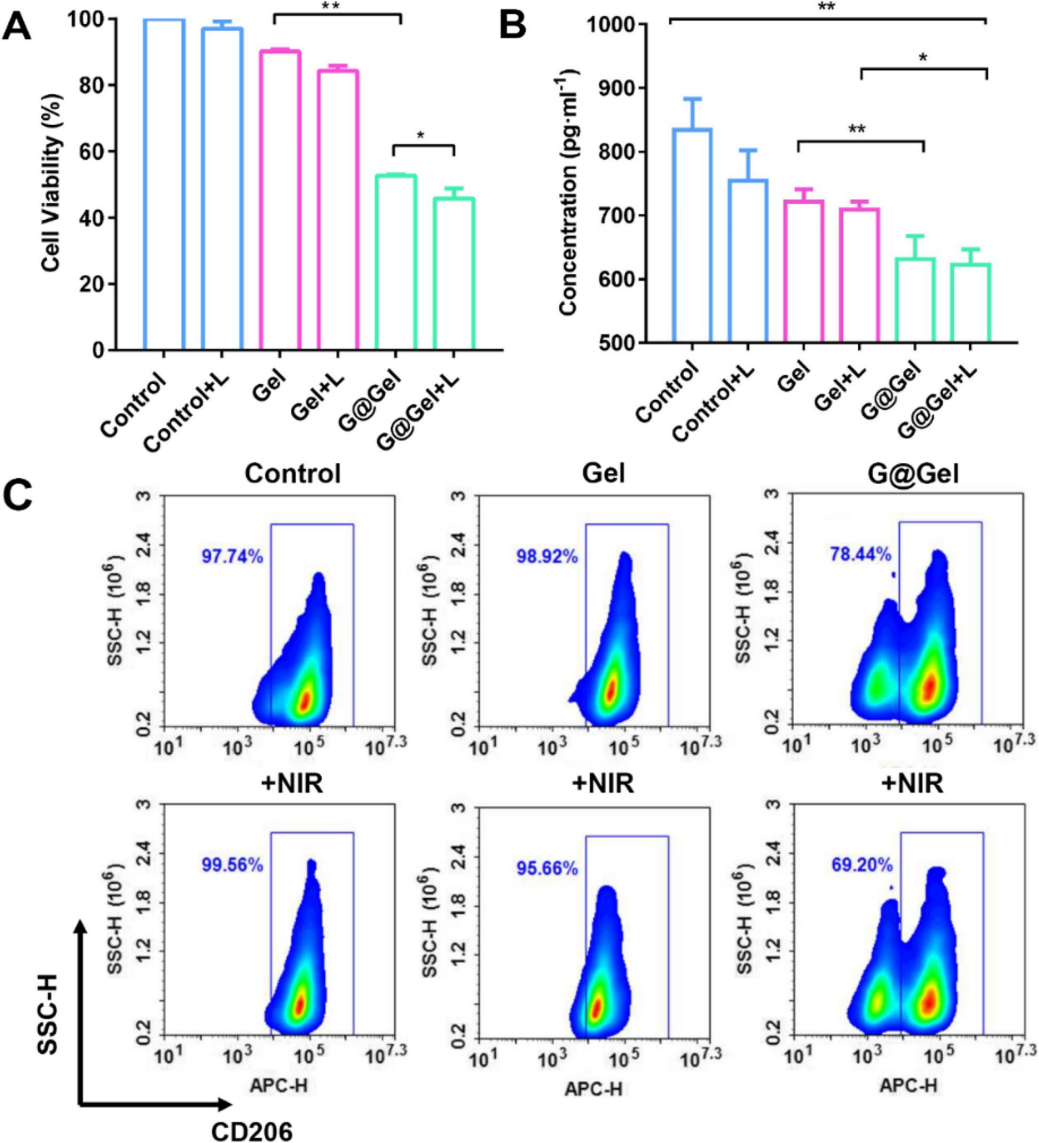
Cytotoxicity of CAFs after various treatments in vitro. **A** The cell viability of CAFs, and (**B**) the concentration of TGF-β in supernatant of CAFs treated with different hydrogels. **C** The differentiation of RAW264.7 after co-incubation with the supernatant of CAFs using flow cytometry after staining with CD206 (Marker for M2 macrophages). Data are shown as mean ± SD (*n* = 3). The comparison of two groups was followed by unpaired Student’s t-test (two-tailed). The level of significance was defined as *p < 0.05, **p < 0.01

The hardness of collagen fibers induced by CAF at the tumor site increased through degradation and redeposition, and the components were not easy to be degraded by matrix metalloproteinases (MMPs), so it was difficult for immune cells and drugs to infiltrate tumor [53]. At the same time, these remodeled collagen fibers by CAF can promote the rapid movement of tumor cells and accelerate tumor invasion [54]. Thus, it is of great importance to decrease CAFs in tumor. The cytotoxicity of G@Gel hydrogel on CAFs was investigated by CCK-8 assay. After treated with TGF-β, the expression of α-SMA and FAP-α in NIH 3T3 cells has increased to form CAFs as in vitro model (Figure S16). As shown in Fig. 4A, the inhibition of CAFs by G@Gel was about 50%, which is significantly higher than that of Gel and Gel with laser (approximately 15%). To figure out the influence of different concentrations of NO on CAFs, we have co-incubated hydrogel containing different concentrations of GSNO in CAF cells. As shown in Figure S17, due to the cytotoxicity of Gel, with low concentrations (0 ~ 0.1 mg/mL), GSNO has no significant toxicity on cell viability of CAF cells. When the concentration reaches a certain level (approximately 0.15 mg/ml), GSNO begins to inhibit CAF cells. Furthermore, compared the cytotoxicity of CAFs with 4T1 cells, without GSNO, the Gel showed toxicity to 4T1 cells, but no significant toxicity to CAFs. When encapsulated GSNO in Gel, G@Gel increased the cytotoxicity to CAFs, which indicated that GSNO could increase the sensitivity of CAFs for CDT. Interestingly, we have found that the inhibition of CAFs by Gel was decreased significantly no matter with or without irradiation compared with that of 4T1 cancer cells, suggesting CAFs may not be sensitive to CDT with ROS generation and PTT by NIR irradiation. To further investigate the uptake of NO by CAFs, DAF-FM DA was used to measure the intracellular content of NO. As expected, only CAFs in G@Gel group had a high concentration of intracellular NO compared with other groups (Figure S18).

### Decrease differentiation of M2 macrophages by inhibiting CAFs secretion of TGF-β

CAF is one of the most important components in TME and plays an essential role in the development of tumor. It has been confirmed to promote the differentiation of monocytes into M2-type macrophages by secreting TGF-β [2]. To evaluate the effect of formed Cu-based hydrogel on secretion of TGF-β in CAFs, the concentration of TGF-β in supernatant of CAFs after treatment was investigated. As shown in Fig. 4B, G@Gel performed the best inhibitory effect on TGF-β secretion, which exhibited a 22% decrease compared with control group. In addition, the secretion of TGF-β only decreased by 8.9% after treated with Gel, revealing an efficient inhibitory effect caused by NO. It is worth noting that NO was reported to decrease myofibroblast differentiation of human keratocytes treated with TGF-β, which performed as down-regulated α-SMA and N-cadherin expression and inhibition of Smad3 phosphorylation [55]. For further investigation, the supernatant of CAFs after treatment was co-incubated with RAW264.7 (Fig. 4C). The differentiation of RAW264.7 in the Gel-treated group was almost the same with the control group, revealing it had no inhibitory effect on the differentiation of RAW264.7 to M2-type macrophages. Besides, after addition of irradiation, the differentiation remains no change, suggesting that CAFs is not sensitive to PTT for the differentiation of M2 macrophages. Interestingly, G@Gel significantly inhibited the differentiation of RAW264.7 to M2-type macrophages, which decreased by nearly 20%, and exhibited a better inhibition after irradiation (69.2% of RAW264.7 were differentiated to M2-type macrophages). It has been reported NO can promote differentiation of macrophages to M1-type macrophages. As shown in Figure S19, GSNO-treated RAW264.7 showed twofold intensity of CD86 compared with control group, indicating GSNO could significantly increase the differentiation of macrophages to M1-type and reverse immunosuppressive TME. All these results indicated that NO plays a significant role in reversing the immunosuppressive TME and enhancing immunotherapy through inhibiting secretion of TGF-β by CAFs.

### Transcriptome analysis of Gel- and G@Gel-treated CAFs

**Fig. 5:**
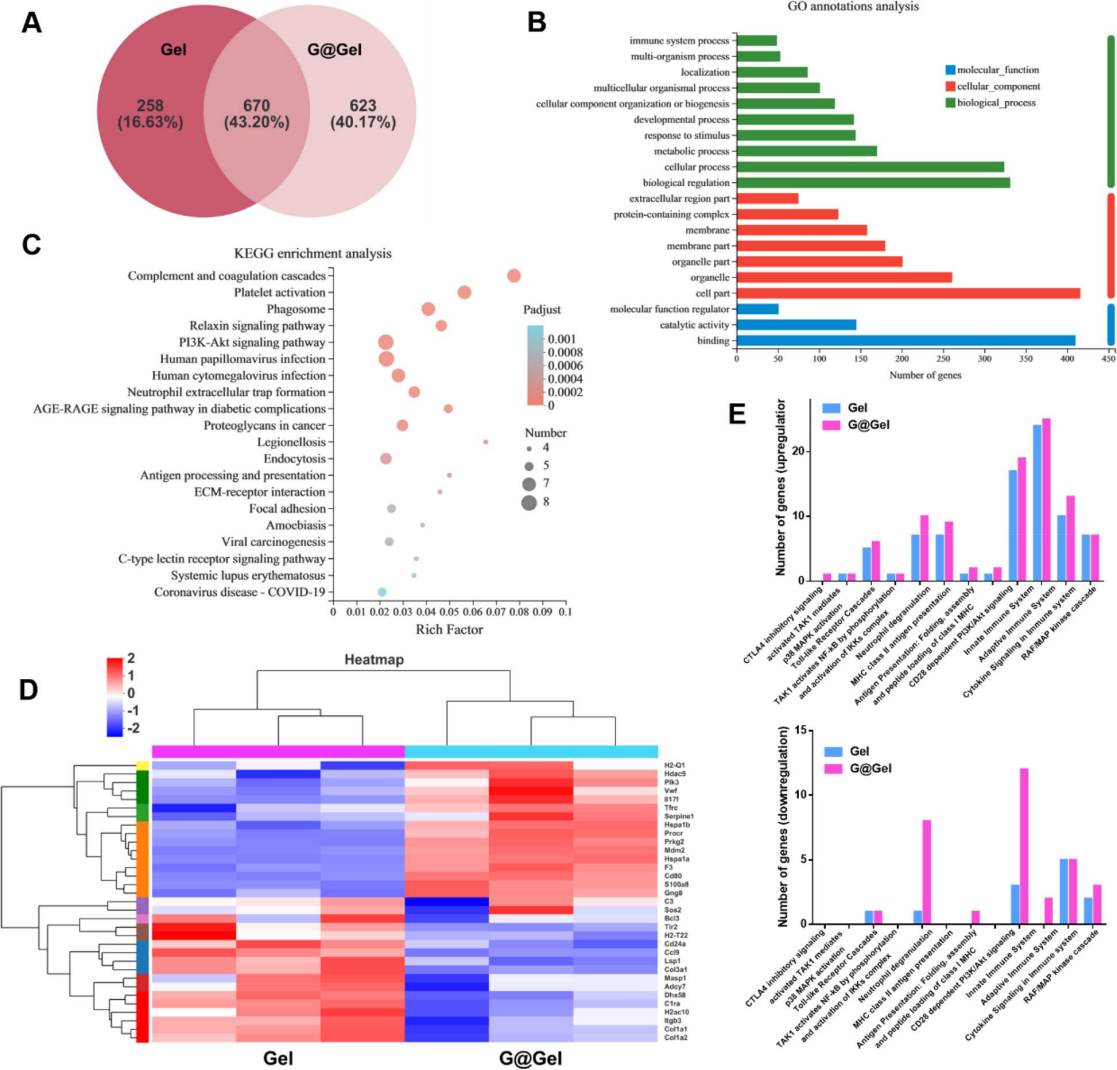
Transcriptome analysis of Gel- and G@Gel-treated CAFs. **A** Venn diagram of a primary transcript between Gel and G@Gel. **B** The differences of genes associated with molecular function, cellular component, and biological process based on GO annotation. **C** KEGG enrichment analysis for immune system associated different genes. **D** Heat map showing significantly upregulated and downregulated genes of immune system after treatment. **E** Upregulated and downregulated genes in different pathways associated with immune system

In order to further investigate the influence of NO on CAFs, we co-incubate Gel and G@Gel with CAFs for 24 h respectively. Then we performed transcriptome analysis on CAFs (the transcriptome analysis was assisted by Majorbio). As shown in Fig. 5A, significant discrepancy of several primary transcripts occurred between Gel-treated and G@Gel-treated groups, 40.17% of genes expressed in G@Gel-treated CAFs were different from that in Gel-treated CAFs. These genes were related to biological process, cellular component and molecular function on the basis of the Gene Ontology (GO) analysis (Fig. 5B). Kyoto Encyclopedia of Genes and Genomes (KEGG) pathway analysis revealed the comprehensive immune response of G@Gel-treated CAFs, in which several major pathways including platelet activation, PI3K-Akt signaling pathway, complement and coagulation cascades and phagosome were activated (Fig. 5C). The cluster analysis showed genes that were significantly differentially expressed in immune system between Gel-treated and G@Gel-treated groups (Fig. 5D), and the analyses of functional interaction network revealed their connection with each other (Figure S20).

The upregulation and downregulation of genes associated with immune system in Gel-treated and G@Gel-treated CAFs are shown in Fig. 5E. Both groups had a number of genes upregulated in CD28 dependent PI3K/Akt signaling, neutrophil degranulation, adaptive immune system and innate immune system. But on the contrary, there were lots of genes downregulated in these pathways in G@Gel-treated CAFs compared with Gel-treated CFAs. Meanwhile, CD28 dependent PI3K/Akt signaling was reported to dictate the activation of T cells [56], which also needs the first signal provided by MHC/ antigenic peptide-TCR. This indicates that NO has effects on the activation of T cells and neutrophils via CAFs and could influence innate and adaptive immune system. Interestingly, Kieffer, Y et al. found that Cluster 0/ecm-myCAF could upregulate PD-1 and CTLA4 protein levels in regulatory T lymphocytes (Tregs) and increase CAF-S1 cluster 3/TGF-β-myCAF cellular content [57]. In our study, G@Gel upregulated more genes in Toll-like receptor cascades than Gel alone. Notably, Toll-like receptor 4 was found to play a key role in advanced glycation end products-induced M1 macrophage polarization [58], indicating that NO may influence the polarization of M1 macrophages and reverse immunosuppressive TME.

### Anti-tumor efficacy in 4T1 tumor-bearing mice

The 4T1 tumor-bearing BALB/c mice were applied to investigate the anti-tumor efficacy of the Cu-induced hydrogel. As proved that Gel could increase the temperature as PTT, so the photothermal conversion ability of hydrogel in vivo was also evaluated by thermal recorder. As shown in Figure S21, the temperature of Gel in tumor site rose to about 45 °C within 60 s after NIR laser irradiation, compared with the control group. The results indicated that the formed hydrogel could show effective photothermal treatment of tumor without inactivating the gel-coated proteins in vivo.

**Fig. 6:**
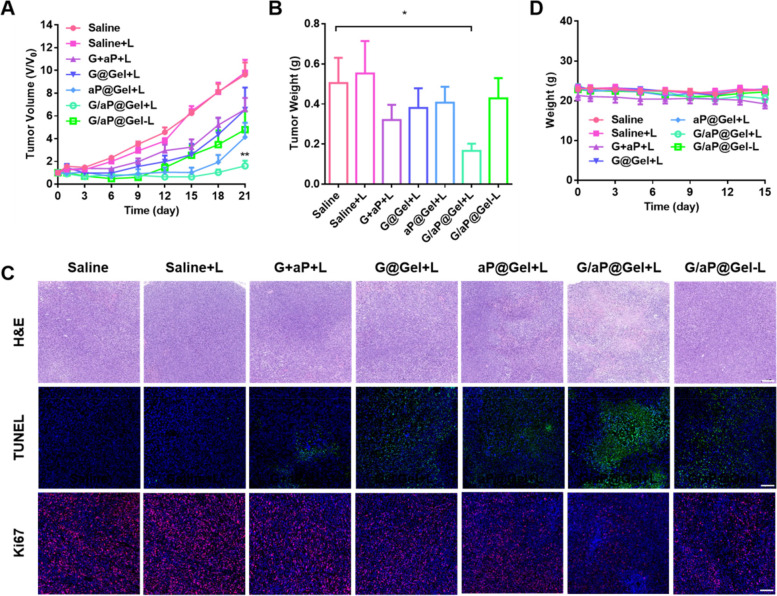
In vivo anti-tumor effects of hydrogel. **A** Growth curves of 4T1 tumor-bearing mice model after various treatments. Data presented as mean ± SEM (*n* = 7). **B** The weight of tumors obtained from the tumor-bearing mice after different treatments. **C** Immunofluorescence staining of H&E and TUNEL in tumors from each group on day 3 after treatment. (H&E, Scale bar = 200 µm, TUNEL, Scale bar = 100 µm, Ki67, Scale bar = 100 µm.). (D) Body weight changes of the mice during the therapy. The comparison of two groups was followed by unpaired Student’s t-test (two-tailed). The level of significance was defined as *p < 0.05, **p < 0.01

Then the anti-tumor efficacy of formed hydrogel was also evaluated in 4T1 tumor-bearing mice. Once the volume of the tumor reached about ~ 200 mm^3^, mice were randomly divided into seven groups and each was treated with saline, G plus aP, G@Gel, aP@Gel and G/aP@Gel, respectively. The NIR irradiation with 808 nm laser was applied to induce PTT for cancer treatment. As shown in the Fig. 6A and Figure S22, it is worth noting that the tumor growth of the saline group with and without light was basically the same, which means that light had no obvious effect on the normal saline group. More important, the G/aP@Gel with laser exhibited an excellent anti-tumor efficacy, which perfectly inhibited tumor growth, and after 21 days treatment its tumor volume is similar to the initial volume. The other groups such as G@Gel with laser, aP@Gel with laser and G/aP@Gel without laser also showed the tumor inhibition in some content. Interestingly, the aP@Gel with L group inhibited tumor growth at the beginning and then showed a quick growth, compared with the treatment of G/aP@Gel with laser. This result indicates that NO, as an immune adjuvant, could induce the death of tumor cells and reverse the tumor microenvironment, which has greatly enhanced tumor treatment.

As described above in vitro, the Cu-based hydrogel could inhibit cancer cells via CDT and PTT. Therefore, G@Gel with laser showed tumor inhibition. However, without combination anti PD-L1, the inhibition rate was only 24.3%, significantly lower than that of G/aP@Gel with laser (76.9%). This result indicated that the G/aP@Gel with laser could induce ICD and increase the infiltration of CTLs, which is responsive for anti PD-L1 for cancer immunotherapy. In addition, without laser irradiation, G/aP@Gel could inhibit tumor growth by the synergistic therapy of CDT and anti PD-L1. The tumor weights of various treatments had been carried out on the day 21 after treatments. As shown in Fig. 6B and Figure S23, the treatment of G/aP@Gel with laser showed the highest inhibition rate compared with other treatments, which is similar as the results of tumor growth. Furthermore, the H&E staining and TUNEL staining of tumors also showed that G/aP@Gel with laser induced the most apoptosis of tumor cells, whose percentage of TUNEL staining was largest (Fig. 6 C and Figure S24A). Other side, Ki67 was the marker indicated the metastasis, which was lower in the treatment of G/aP@Gel with laser (Figure S24B). All these results indicated that the formed hydrogel with GSNO and anti PD-L1 could significantly inhibit tumor growth with the combination of PTT and CDT, which enhance immunotherapy. The weight of each group had almost no change, suggesting none systemic toxicity occurred within 15 days (Fig. 6D). The H&E staining of main organs (including the liver, lung, kidney, spleen and heart), which presented almost the same, further confirmed that there was no damage in the mice during treatments (Figure S25).

### The reverse of immunosuppressive TME by interfering hydrogel with CAFs in vivo

**Fig. 7:**
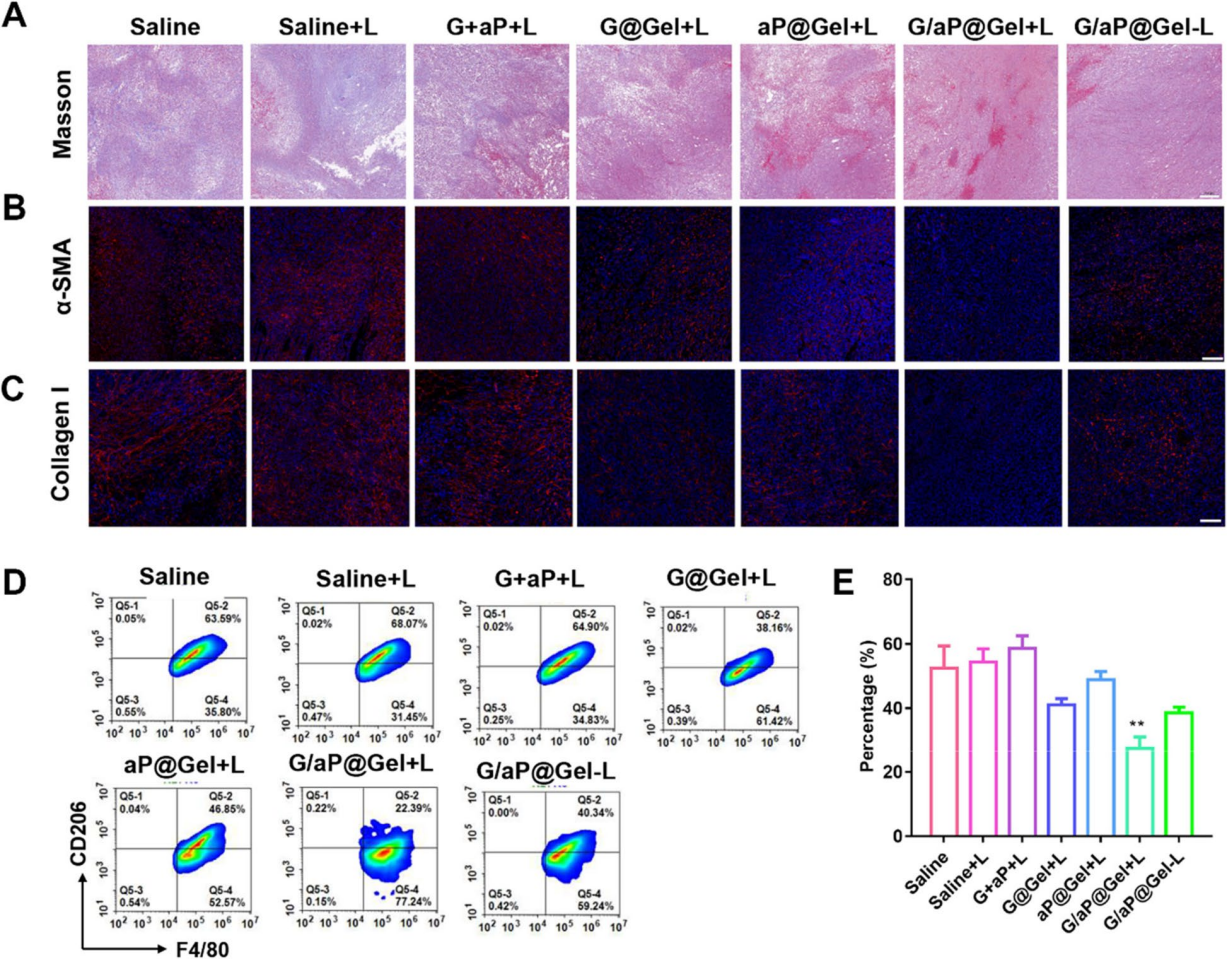
Reduction of M2-type macrophages in tumor through the treatment of hydrogel by interfering with CAFs. **A** Masson’s trichrome staining of collagen, (Scale bar = 200 µm) (**B**) immunofluorescence staining of α-SMA (Scale bar = 100 µm) and (**C**) collagen I (Scale bar = 100 µm) in tumors after various treatments. **D** Representative flow cytometry plots showing percentages of M2-type macrophages in tumors on day 5 after treatments. **E** The percentages of M2-type macrophages in tumors upon various treatments. Data presented as mean ± SD (*n* = 5). The comparison of two groups was followed by unpaired Student’s t-test (two-tailed). The level of significance was defined as *p < 0.05, **p < 0.01

As proved that, in vitro, the release of NO could significantly increase the sensitivity of CAFs for CDT and PTT, and thus reduce the differentiation of M2 macrophages. Therefore, to figure out the effects of our hydrogel on CAFs in vivo, Masson’s trichrome staining of collage, immunofluorescence staining of α-SMA and collagen I staining on the tumor tissues have been evaluated on day 5 after various treatments. As shown in Fig. 7A and Fig. 7B, G/aP@Gel with laser could significantly reduce the content of collagen fiber and collagen I at the tumor site. At the same time, the expression of α-SMA (main marker of CAFs) in the G/aP@Gel with laser group decreased significantly as well (Fig. 7C). Interestingly, compared the red fluorescence of α-SMA and collagen I in G/aP@Gel with or without laser (Figure S26 and S27), the results indicated that the photothermal of PTT could also reduce the CAFs via hyperthermia. In addition, compared with aP@Gel plus laser, the expression of α-SMA in G/aP@Gel was lower, which indicated that NO could induce the depletion of CAFs. Therefore, considering the relationships between CAFs and ECM, all the results reveled that G/aP@Gel with laser could reverse TME through interfering with CAFs.

For further investigation, the population of M2-type macrophages in tumor issues was evaluated by flow cytometry. As shown in Fig. 7D and E, the percentage of M2-type macrophages in G/aP@Gel with Laser-treated group dropped by nearly half compared with Saline-treated group. In addition, all the groups treated with hydrogel all had a decrease in percentage of M2-type macrophages. The percentage of M2-type macrophages in aP@Gel with laser dropped about 20% compared with saline. The fluorescence staining of M2 macrophages and M1 macrophages were shown in Figure S28 and S29. The result was similar as determination of flow cytometry. It may be due to the photothermal effect of CAFs, which is consistent with the staining of CAFs above. The hydrogel with NO release could significantly reduce the M2 macrophage and the results indicated that the NO generation could regulate M2 macrophages by interfering with CAFs. The staining images of TGF-β in tumor issues further confirmed that hydrogel could reduce the secretion of TGF-β in tumor, in which G/aP@Gel with laser caused the largest decrease (Figure S30 and S31). These results reveal that G/aP@Gel with laser could decrease the secretion of TGF-β by depletion of CAFs and thereby decrease the percentage of M2-type macrophages and increase the percentage of M1-type macrophages in tumor.

### Amplifying ICD to enhance immunotherapy by hydrogel

**Fig. 8:**
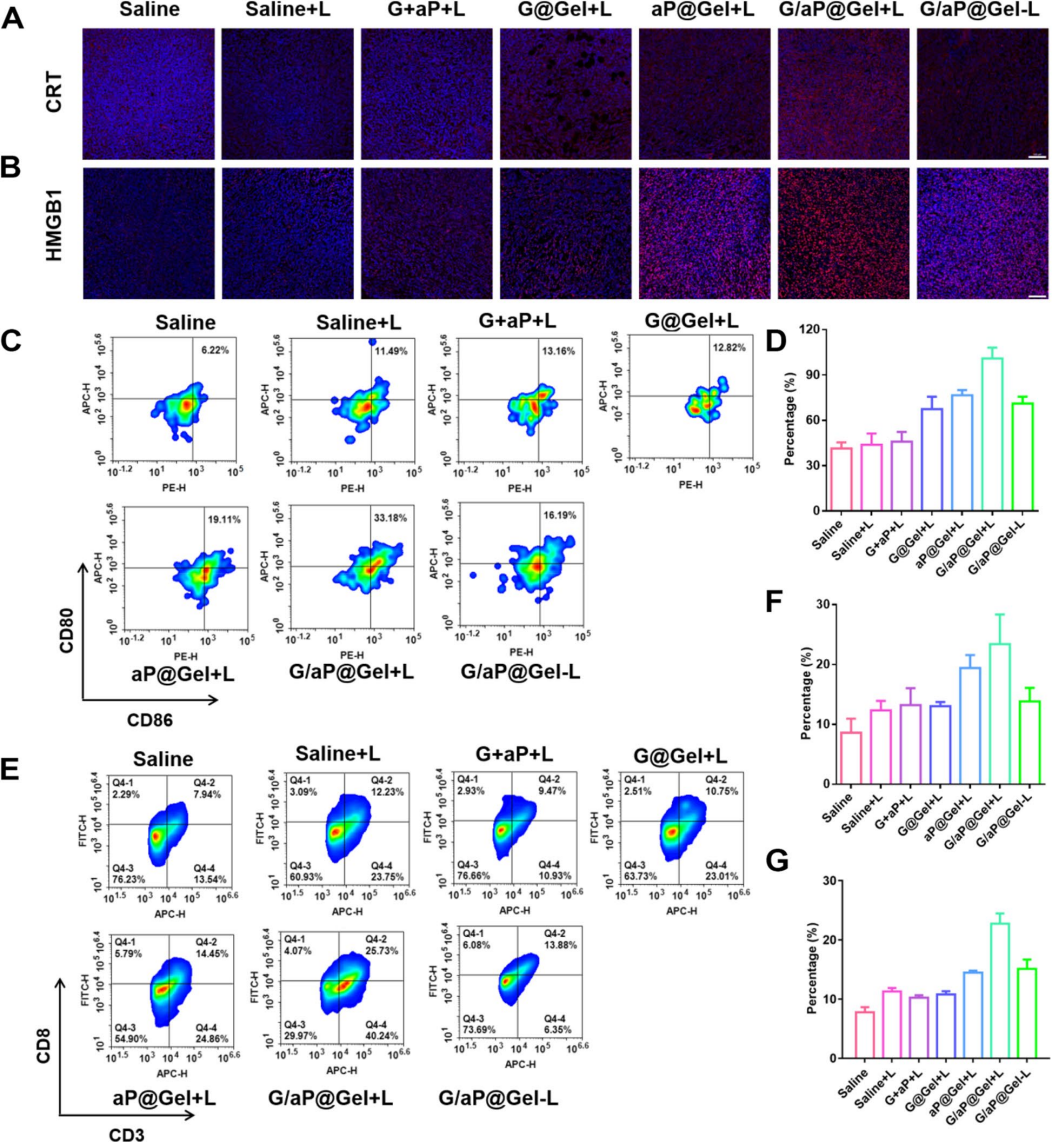
Anti-tumor immune response induced by hydrogel. Immunofluorescence staining of (**A**) CRT and (**B**) HMGB1 in tumors after various treatments, Scale bar = 100 µm. **C** Representative percentages of matured DCs on day 5 after different treatments by flow cytometry. **D** Relative CRT level with immunofluorescence staining. **E** Flow cytometric examination of the intratumor infiltration of CD8^+^T cells after various treatments. **F** The percentage of matured DCs in lymph nodes. **G** The relative CD8^+^T cells frequencies in tumors with the analysis of flow cytometry. Data presented as mean ± SD (*n* = 5). The comparison of two groups was followed by unpaired Student’s t-test (two-tailed). The level of significance was defined as *p < 0.05, **p < 0.01

Beside the immunosuppressive TME, ICD is the key inducer for cancer immunotherapy. It has been proved above that the synergetic therapy of Gel could amplify ICD by expressing high level of CRT and HMGB1 and releasing more ATP in vitro. In order to investigate the ICD in vivo, the immunofluorescence staining of CRT and HMGB1 have been evaluated in tumors after various treatments. As shown in Fig. 8A and B, G/aP@Gel with laser showed the bright red fluorescence, which indicated that formed hydrogel could significantly increase the level of CRT and HMGB1 in tumors. The comparison of the fluorescence intensity of CRT and HMGB also showed that our strategy could effectively induce ICD of tumor cells, which could eventually activate the anti-tumor immune responses (Fig. 8D and Figure S32).

As known to us, DCs are the most functional professional antigen presenting cells for cancer immunotherapy [59]. They are equivalent to messenger, which transmits antigen information to T cells and therefore activate T cells [60]. To figure out the anti-tumor immune response caused by our strategy, the maturation of DCs in lymph nodes had been evaluated by flow cytometry. As shown in Fig. 8C and F, G/aP@Gel with laser induced most percentage of DCs maturation in tumor as expected, which is 2.72-, 1.79- and 1.69-fold relative to Saline, G@Gel with laser and G/aP@Gel without laser, respectively. This result supported that the amplified ICD could increase the maturation of DCs for anti-tumor immune response. With the reverse of immunosuppressive TME of amplifying ICD of tumors, the proportion of CD8^+^T cells was last investigated, as shown in Fig. 8E, G and Figure S33. Due to the released anti PD-L1, the hydrogel with ICB showed more infiltration than other groups. Although the NO from G@Gel could reverse the immunosuppressive TME, but we found that the G@Gel showed less infiltration of CD8^+^T cells. This may be due to the lower ICD to active anti-tumor immune response. However, the proportion of CD8^+^T cells in G/aP@Gel with laser treated group is more than twice as the other groups. Furthermore, G/aP@Gel + L could significantly increase the secretion of TNF-α in tumors (Figure S34 and S35), revealing it could enhance anti-tumor immune responses. All these results indicated that our formed hydrogel with GSNO and anti PD-L1 could successfully activate immune responses and increase the infiltration of cytotoxic T cells in tumors by reversing the TEM and amplifying ICD.

A bilateral tumor model was also established to investigate whether the immunohydrogel had a strong enough immune response to inhibit the untreated distant tumor. As shown in Figure S36A, the inhibitory effect of each group on in situ tumor was consistent with the previous experimental results. Meanwhile, G/aP@Gel with laser also showed superior inhibitory effect on distant tumors (Figure S36B). Compare with treated group of Saline with laser, whose tumor size increased to 1400 mm^3^, tumors in G/aP@Gel with laser only increased to about 300 mm^3^, which is similar to its original size. These results proved that our formed hydrogel could induce a strong systemic immune response in mice, therefore inhibiting the growth of distant tumor without treatment. The weight of mice in each group during the treatment remained none change (Figure S37).

### Safety evaluation of hydrogel in vivo

**Fig. 9:**
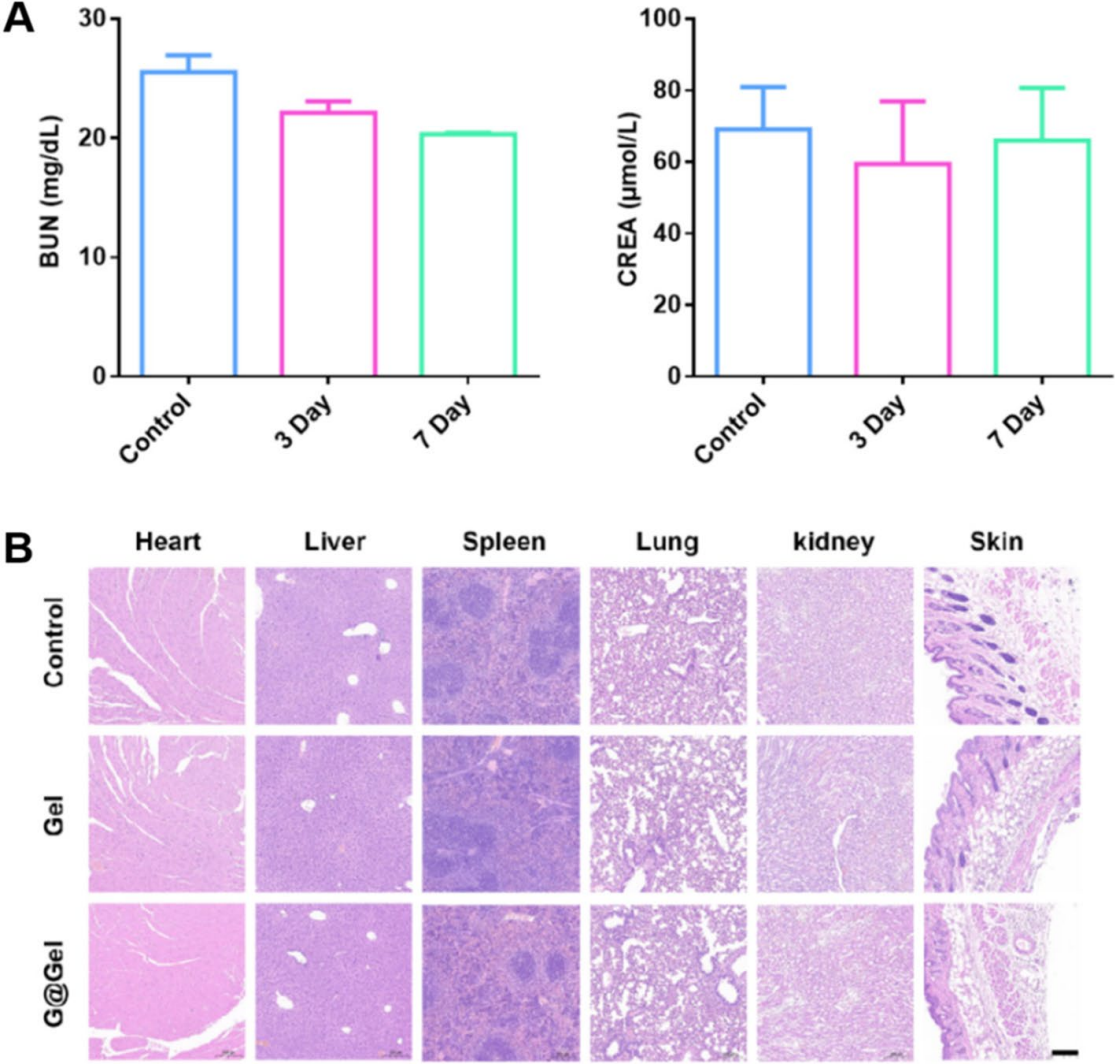
Safety evaluation of hydrogel in healthy mice. **A** BUN and CREA level in serum after treatment of G@Gel for 3 and 7 days. **B** H&E staining of main organs in healthy mice after 7 days treatment, Scale bar = 200 µm

The blank hydrogel was subcutaneously injected into normal mice (GSNO, 0.2 mg·mL^−1^; CuCl_2_, 0.92 mg·mL^−1^), and the mice were killed on the third and seventh day after treatment. Their main organs, skin and serum were then collected and detected. As shown in Fig. 9A, there was no significant difference in the concentration of BUN and CREA between the treated group and the control group. The H&E staining of heart, liver, spleen, lung, kidney and skin showed that hydrogel had no physiological toxicity to normal mice as well (Fig. 9B). All these results indicated that our designed hydrogel had a high biocompatible characteristic and had potential application for cancer immunotherapy.

## Discussion

As stromal cells, cancer-associated fibroblasts (CAFs) play a crucial role in the tumor microenvironment (TME). CAFs secrete cytokines such as TGF-β, IL-6, and CCL2, which promote the recruitment of monocytes and their differentiation into M2-type tumor-associated macrophages (TAMs). This process impairs the response of efferent T cells and induces immunosuppression in TME. In addition to facilitating macrophage recruitment and trans-differentiation, CAFs induce the immunosuppressive properties of TAMs [2]. Recent studies have shown that CAF-induced M2-type TAMs express high levels of programmed death 1 (PD-1) [61]. They have also confirmed that high level of PD-1 expression of TAMs suppresses innate and adaptive anti-tumor immune responses, including reducing their own phagocytic ability against tumor cells and inhibiting the infiltration and proliferation of T cells [62]. These results suggest that CAFs are responsible for the immunosuppression of TAMs.

Conversely, M2-type TAMs have been reported to enhance the process of epithelial-mesenchymal transition (EMT) by secreting soluble factors such as IL-6 and SDF-1, stimulating the activation of CAFs. This indicates that CAFs and TAMs have positive feedback effects on each other in promoting tumor development. Recent studies have shown that NO produced by M2-type TAMs protects tumor cells from cisplatin, suggesting that endogenous NO production is the main mechanism by which TAMs protect tumor cells from chemotherapy [63]. Meanwhile, CAFs can increase intracellular NO secretion by expressing a large number of iNOS. This induces tumor cells to increase the secretion of chemotherapy resistance inducer IL-1β, thus promoting the development of tumor chemotherapy resistance [64]. These results suggest that NO plays an important role in regulating TAMs and CAFs and promoting tumor resistance. Our study found that NO can deplete tumor cells in a dose-dependent manner. Moreover, low doses of NO had no significant toxicity on the viability of CAFs, but higher concentrations inhibited CAFs. Furthermore, NO promoted the polarization of monocytes to M1-type macrophages. Several studies have reported the significant therapeutic effect of high concentrations of NO on tumors [33, 34, 37], suggesting the great potential of NO in regulating TAMs and CAFs and enhancing tumor therapy.

Previous have reported that most nanoparticles have defects such as short storage in vivo, low bioavailability, unstable drug release, and high systemic toxicity [65]. In addition, the NO donor delivered by nanoparticles is easily to release due to the complex biological environment in vivo, resulting in systemic toxicity and low therapeutic effect. Injectable hydrogels have been widely used in the encapsulation of various bioactive drugs for cancer treatment. They have specific advantages to release drugs in targeted tissues, resulting in low systemic toxicity, controllable and sustained drug release and significant efficacy. Delivery of NO via hydrogel can significantly improve the storage of NO donor in vivo and increase its bioavailability. Hydrogel can also prevent NO donor from abrupt release induced by external factors through avoiding circulation in vivo. The copper ions contained in hydrogel can perform photothermal conversion under near-infrared irradiation to achieve controlled rise of temperature, which promotes the release of NO and provides sufficient gas for treatment. Besides, exogenous HA was reported to competitively bound to CD44 with endogenous HA, which leads to the suppression of PI3-kinase/Akt cell survival pathway and consequently to inhibition of anchorage-independent growth in culture and tumor growth in vivo [66], indicating hydrogel made from HA has great potential in tumor treatment.

## Conclusion

In summary, the biocompatible injectable hydrogel was successfully developed via the chelation of Cu ions and sulfhydryl groups to encapsulate GSNO and anti PD-L1 for cancer immunotherapy. Due to the NIR absorption of Cu ions, the formed hydrogel could increase temperature with hyperthermia as PTT. As evaluated in vitro, due to the high expression of GSH and H_2_O_2_ in tumor microenvironment, the formed Gel could release Cu^2+^ and reduce to Cu^+^ with GSH, thereby generation ·OH as CDT. The GSH could induce Gel to degrade to release anti PD-L1 for cancer treatment. Furthermore, the GSH and increased temperature could also increase NO release from GSNO. The formed Gel could significantly kill 4T1 tumor cells by the synergistic therapy of PTT, CDT and NO gas. The released CRT, HMGB1 and ATP was amplified to mature the DC for antigen presentation. Moreover, we found that NO could interfere with CAFs to inhibit its cell viability and decrease its secretion of TGF-β, thus reducing the differentiation M0 to the M2 macrophages. The transcriptome analysis revealed the NO may regulate the genes expression of PI3K/Akt signaling and Toll-like receptor cascades, which interfaced with CAFs to reverse immunosuppressive TME. After carried out the anti-tumor study on 4T1 tumor-bearing mice, the formed hydrogel could also significantly inhibit tumor growth. It could increase the infiltration of CTLs and reduce the percentages of M2 macrophages for enhanced cancer immunotherapy, due to the amplified of ICD to mature DC and reversing immunosuppressive TME by depletion of CAFs. This study provides a novel strategy with ion-induced hydrogel with NO for cancer immunotherapy via regulating CAFs in immunosuppressive TME.

## Supplementary Information

**Additional file 1:** **Figure S1.** Infrared spectrogram of HA and HA-SH. **Figure S2.** Detection of -SH through the reduction of DTNB to TNB. **Figure S3.** SEM image of interior of the hydrogel. Scale bar=1mm. **Figure S4.** The degradation of hydrogel in vivo. **Figure S5.** Heating and cooling curves of hydrogel for five cycles by turning on and off laser. **Figure S6.** Images of hydrogel after irradiation. **Figure S7.** Release curve of NO from hydrogel in solution added with GSH. Data presented as mean ± SD. **Figure S8.** Detection of Cu^+^ generation with GSH addition using neocuproine hydrochloride monohydrate. **Figure S9.** Release curve of NO from G@Gel without NIR irradiation. **Figure S10.** The degradation behavior of hydrogel under different conditions. **Figure S11.** MB degradation rate under different concentration of CuCl_2_ at different time. **Figure S12.** The generation of ·OH under different concentrations of H_2_O_2_ determined by oxidized TMB. **Figure S13.** Detection of ROS generation in 4T1 tumor cells by staining with DCFH-DA. Scale bar=50µm. **Figure S14.** Cell viability of 4T1 cells treated with different concentration of CuCl_2_ and GSNO. Data presented as mean ± SD. **Figure S15.** Relative CRT level in 4T1 cells after various treatments. The comparison of two groups was followed by unpaired Student’s t-test. The level of significance was defined as *p < 0.05, **p < 0.01. **Figure S16.** Images of immunofluorescence staining ofFAP-α andα-SMA in NIH 3T3 cells after treated with TGF-β, Scale bar=50µm. **Figure S17.** Cell viability of CAF after co-incubation with hydrogel containing different concentrations of GSNO for 24 hours. **Figure S18.** Uptake of NO by CAFs using DAF-FM DA to detective intracellular NO, Scale bar=50µm. **Figure S19.** The differentiation of RAW264.7 to M1-type macrophages induced by GSN. RAW264.7 were stained with CD86. Data are shown as mean ± SD. **Figure S20.** The analyses of functional interaction network of G@Gel regulated genes. **Figure S21.** Temperature curve of the hydrogel irradiated with 808nm in 4T1 tumor-bearing mice. Data presented as mean ± SD. **Figure S22.** Growth curves of tumors in each treatment. **Figure S23.** Photographs of tumors obtained from mice after 21 days treatment. **Figure S24.** RelativeTUNEL andKi67 level in tumor immunofluorescence staining after various treatments. The comparison of two groups was followed by unpaired Student’s t-test. The level of significance was defined as *p < 0.05, **p < 0.01. **Figure S25.** H&E staining of main organs on day 5 after various treatments, Scale bar=500µm. **Figure S26.** Relative α-SMA level in tumor immunofluorescence staining after various treatments. The comparison of two groups was followed by unpaired Student’s t-test. The level of significance was defined as *p < 0.05, **p < 0.01. **Figure S27**. Relative Collagen I level in tumor immunofluorescence staining after various treatments. The comparison of two groups was followed by unpaired Student’s t-test. The level of significance was defined as *p < 0.05, **p < 0.01. **Figure S28.** Images of immunofluorescence staining of M2 macrophages. DAPI, Blue; CD206, Red. Scale bar=50µm. **Figure S29.** Images of immunofluorescence staining of M1 macrophages. DAPI, Blue; F4/80, Red; CD86, Green. Scale bar=50µm. **Figure S30.** Images of immunofluorescence staining of TGF-β in different treatments, Scale bar=100µm**. Figure S31.** Relative TGF-β level in tumor immunofluorescence staining after various treatments. The comparison of two groups was followed by unpaired Student’s t-test. The level of significance was defined as *p < 0.05, **p < 0.01. **Figure S32.** Relative HMGB1 level in tumor immunofluorescence staining after various treatments. **Figure S33.** Images of immunofluorescence staining of T cells in tumor. DAPI, Blue; CD3, Green; CD4, Red; CD8, Pink. Scale bar=50µm. **Figure S34.** Images of immunofluorescence staining of TNF-α in tumor after various treatments, Scale bar=100µm. **Figure S35.** Relative TNF-α level in tumor fluorescence staining after various treatments. The comparison of two groups was followed by unpaired Student’s t-test. The level of significance was defined as *p < 0.05, **p < 0.01. **Figure S36.** Growth curve ofprimary tumor anddistant tumor after treatments. **Figure S37.** Body weight of each group with different treatments.

## Data Availability

The datasets used and/or analyzed during the current study are available from the corresponding author on reasonable request.

## Abbreviations

IT: Immunotherapy
CDT: Chemodynamic therapy
PTT: Photothermal therapy
RT: Radiation therapy
CT: Chemotherapy
PDT: Photodynamic therapy
GT: Gas therapy
CAF: Cancer associated fibroblast
TME: Tumor microenvironment
ATP: Adenosine triphosphate
CRT: Calreticulin
PD-1: Programmed death 1
PD-L1: Programmed death ligand 1
CTL: Cytotoxic T lymphocyte
TGF-β: Transforming growth factor-β
IL-6: Interleukin-6
GSNO: S-Nitrosoglutathione
DTT: DL-Dithiothreitol
HA: Hyaluronic acid
DAMP: Damage associated molecular pattern
NO: Nitric oxide
TAM: Tumor associated macrophage
DAPI: 2-(4-Amidinophenyl)-6-indolecarbamidine dihydrochloride
H_2_O_2_: Hydrogen peroxide
DC: Dendritic cell
EDC: 1-Ethyl-3-(3ʹ-dimethylaminopropyl) carbodiimide
FITC: Fluorescein isothiocyanate
ECM: Extracellular matrix
MMP: Matrix metalloproteinase
DCFH-DA: 2,7-Dichlorodihydrofluorescein acetoacetic acid
H&E: Hematoxylin and eosin
HMGB1: High mobility group box protein 1
ICB: Immune checkpoint blockade
ICD: Immunogenic cell death
MFI: Median fluorescence intensity
NHS: N-hydroxy succinimide
·OH: Hydroxyl radical
PBS: Phosphate buffer saline
ROS: Reactive oxygen species
SEM: Scanning electron microscope
TMB: 3,3ʹ,5,5ʹ-Tetramathylbenzidine
MB: Methylene blue
TUNEL: Terminal deoxynucleotidyl transferase dUTP nick end labeling
HSA: Human serum albumin
Cu: Copper
GSH: Glutathione
EMT: Epithelial-mesenchymal transition
