# Supplementary material for: Copper-induced injectable hydrogel with nitric oxide for enhanced immunotherapy by amplifying immunogenic cell death and regulating cancer associated fibroblasts
Source: Biomater Res. 2023 May 10;27:44. doi: 10.1186/s40824-023-00389-4 (PMC10170699; doi:10.1186/s40824-023-00389-4)
Supplement: Supplementary file 1 — Additional file 1: Figure S1. Infrared spectrogram of HA and HA-SH. Figure S2. Detection of -SH through the reduction of DTNB to TNB. Figure S3. SEM image of interior of the hydrogel. Scale bar=1mm. Figure S4. The degradation of hydrogel in vivo. Figure S5. Heating and cooling curves of hydrogel for five cycles by turning on and off laser. Figure S6. Images of hydrogel after irradiation. Figure S7. Release curve of NO from hydrogel in solution added with GSH. Data presented as mean ± SD. Figure S8. Detection of Cu+ generation with GSH addition using neocuproine hydrochloride monohydrate. Figure S9. Release curve of NO from G@Gel without NIR irradiation. Figure S10. The degradation behavior of hydrogel under different conditions. Figure S11. MB degradation rate under different concentration of CuCl2 at different time. Figure S12. The generation of ·OH under different concentrations of H2O2 determined by oxidized TMB. Figure S13. Detection of ROS generation in 4T1 tumor cells by staining with DCFH-DA. Scale bar=50µm. Figure S14. Cell viability of 4T1 cells treated with different concentration of CuCl2 and GSNO. Data presented as mean ± SD. Figure S15. Relative CRT level in 4T1 cells after various treatments. The comparison of two groups was followed by unpaired Student’s t-test. The level of significance was defined as *p < 0.05, **p < 0.01. Figure S16. Images of immunofluorescence staining ofFAP-α andα-SMA in NIH 3T3 cells after treated with TGF-β, Scale bar=50µm. Figure S17. Cell viability of CAF after co-incubation with hydrogel containing different concentrations of GSNO for 24 hours. Figure S18. Uptake of NO by CAFs using DAF-FM DA to detective intracellular NO, Scale bar=50µm. Figure S19. The differentiation of RAW264.7 to M1-type macrophages induced by GSN. RAW264.7 were stained with CD86. Data are shown as mean ± SD. Figure S20. The analyses of functional interaction network of G@Gel regulated genes. Figure S21. Temperature curve of the hydrogel irradiated wit [file 40824_2023_389_MOESM1_ESM.docx]

**Copper-induced Injectable Hydrogel with Nitric Oxide for Enhanced Immunotherapy by Amplifying Immunogenic Cell Death and Regulating Cancer Associated Fibroblasts**

Shuilin Shen, Zimeng Zhang, Haixiao Huang, Jing Yang, Xinyue Tao, Zhengjie Meng, Hao Ren*, Xueming Li*

School of Pharmaceutical Science, Nanjing Tech University, Nanjing, Jiangsu 211816, China

**Figure S1.** Infrared spectrogram of HA and HA-SH.

**Figure S2**. Detection of -SH through the reduction of DTNB to TNB.


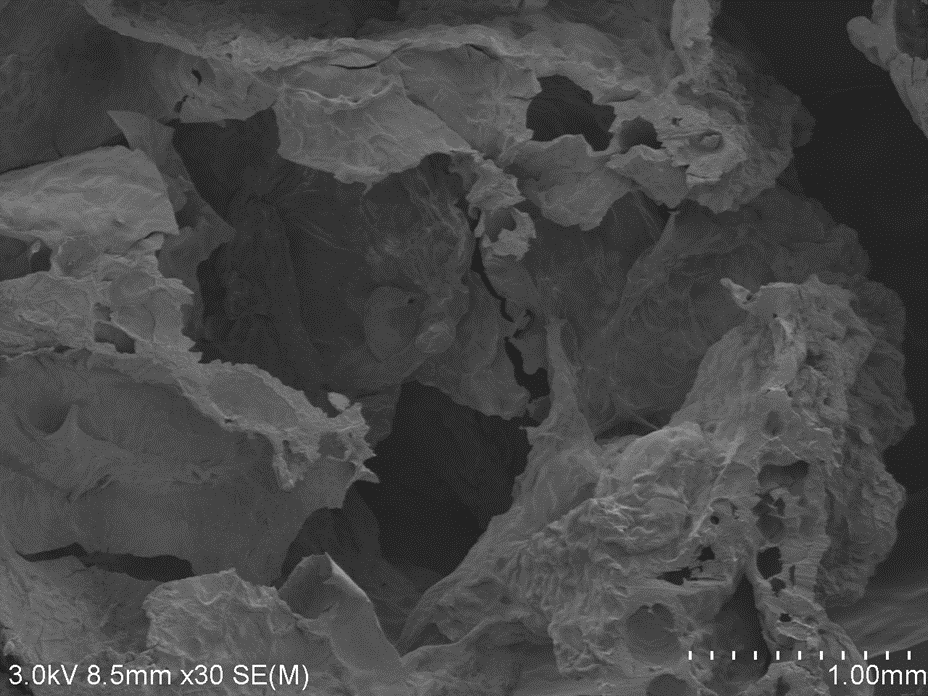


**Figure S3.** SEM image of interior of the hydrogel. Scale bar=1mm.


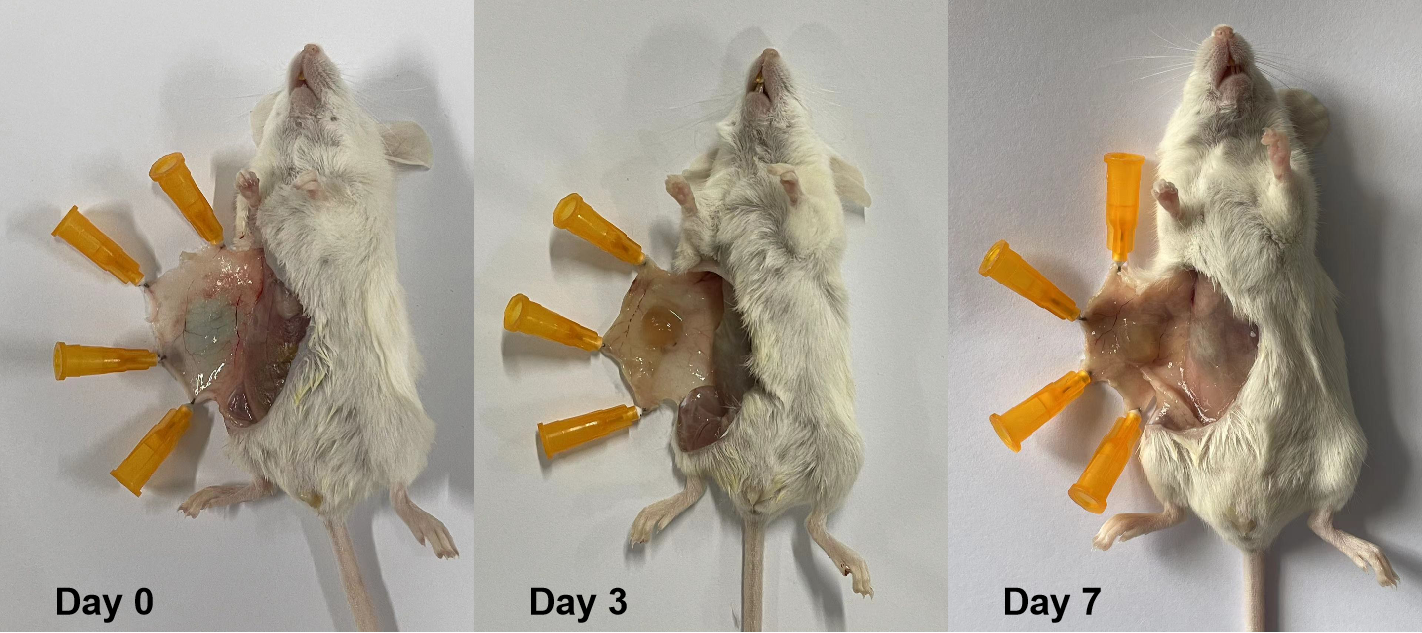


**Figure S4.** The degradation of hydrogel in vivo.

**Figure S5.** Heating and cooling curves of hydrogel for five cycles by turning on and off laser.


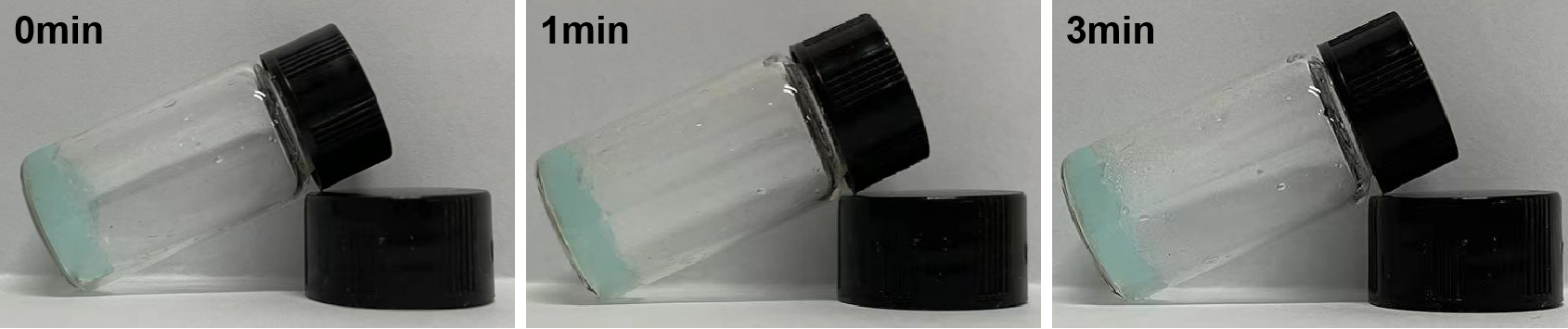


**Figure S6**. Images of hydrogel after irradiation.

**Figure S7.** Release curve of NO from hydrogel in solution added with GSH. Data presented as mean ± SD (n = 3).


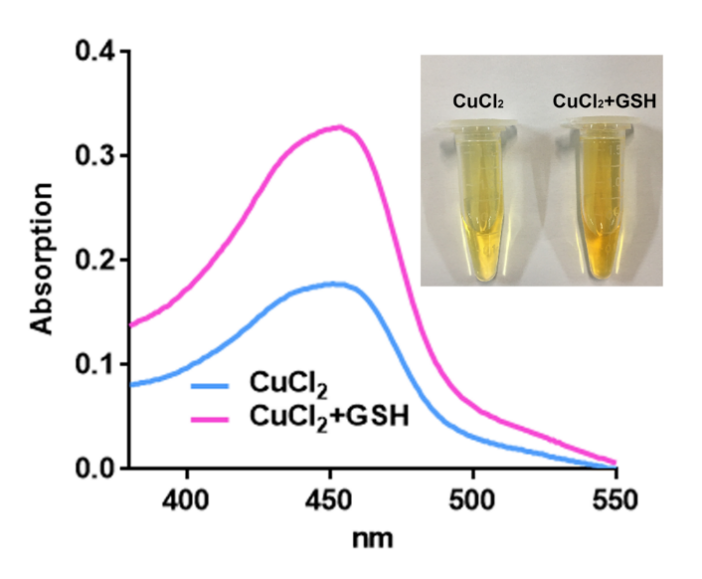


**Figure S8.** Detection of Cu^+^ generation with GSH addition using neocuproine hydrochloride monohydrate.

**Figure S9**. Release curve of NO from G@Gel without NIR irradiation.


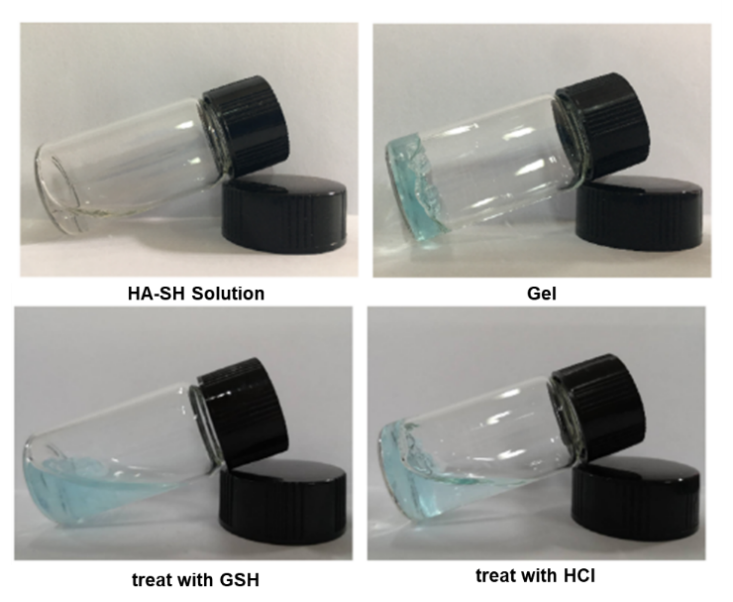


**Figure S10.** The degradation behavior of hydrogel under different conditions.


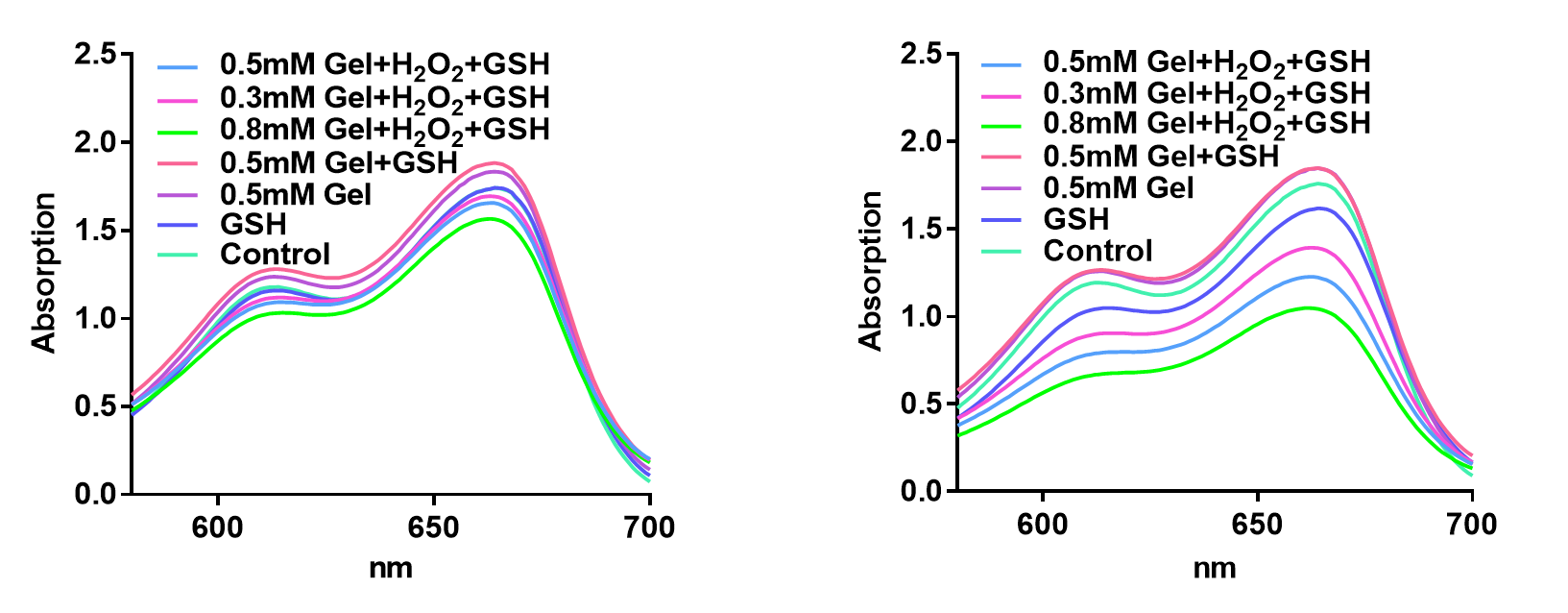


**Figure S11.** MB degradation rate under different concentration of CuCl_2_ at different time (left, 4h, right, 19h).

**Figure S12.** The generation of ·OH under different concentrations of H_2_O_2_ determined by oxidized TMB.


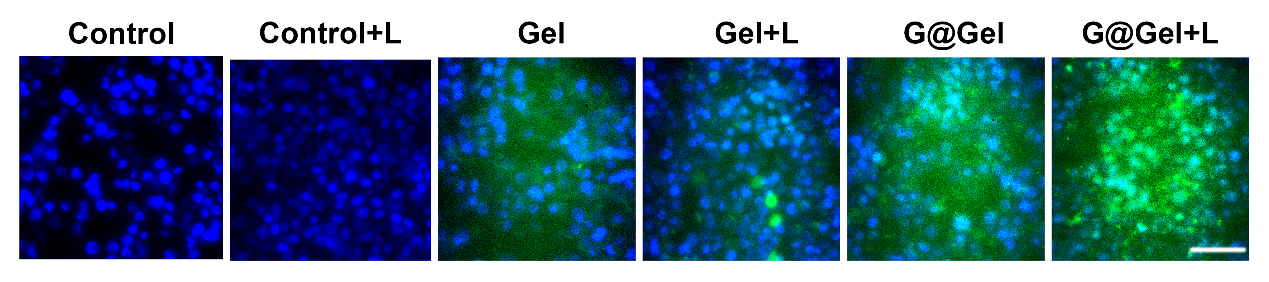


**Figure S13.** Detection of ROS generation in 4T1 tumor cells by staining with DCFH-DA. Scale bar=50µm.


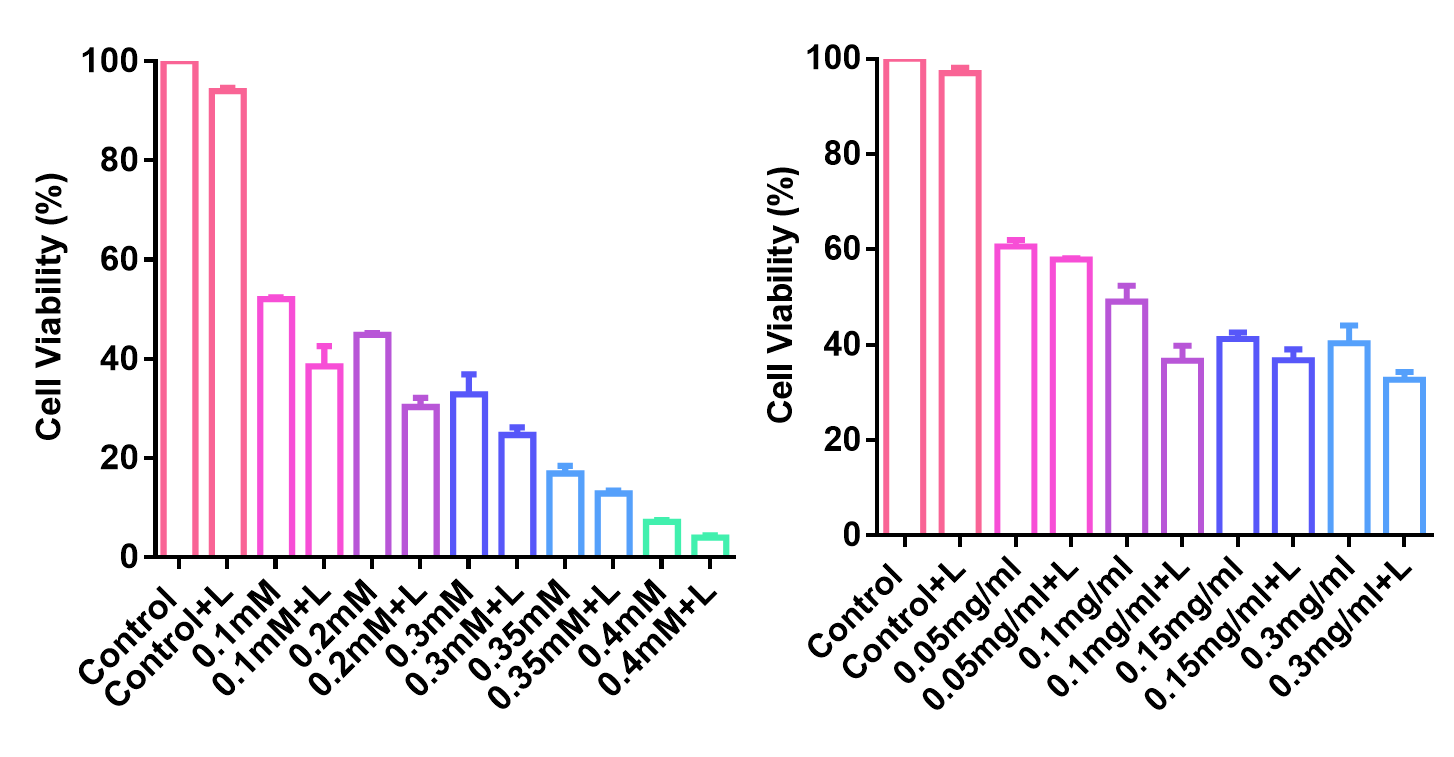


**Figure S14.** Cell viability of 4T1 cells treated with different concentration of CuCl_2_ and GSNO (left, CuCl_2_; right, GSNO). Data presented as mean ± SD (n = 3).


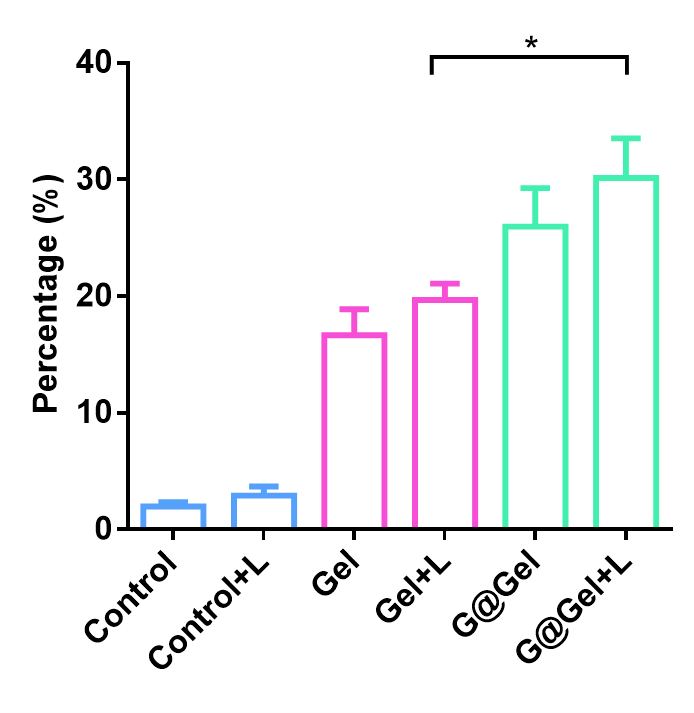


**Figure S15.** Relative CRT level in 4T1 cells after various treatments. The comparison of two groups was followed by unpaired Student’s t-test (two-tailed). The level of significance was defined as *p < 0.05, **p < 0.01.


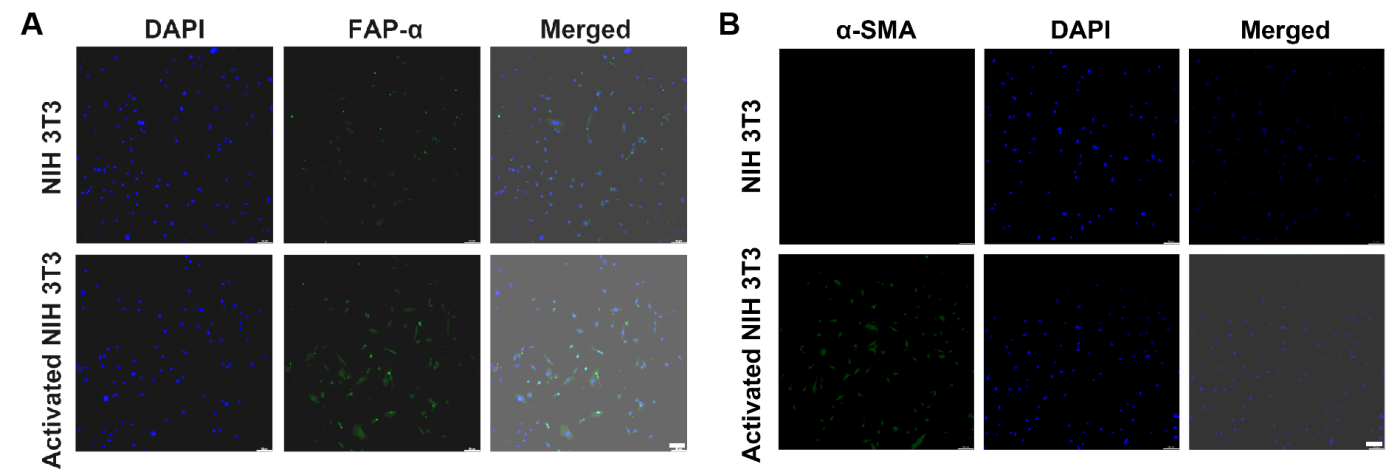


**Figure S16**. Images of immunofluorescence staining of (**A**) FAP-α and (**B**) α-SMA in NIH 3T3 cells after treated with TGF-β, Scale bar=50µm.

**Figure S17**.Cell viability of CAF after co-incubation with hydrogel containing different concentrations of GSNO for 24 hours.


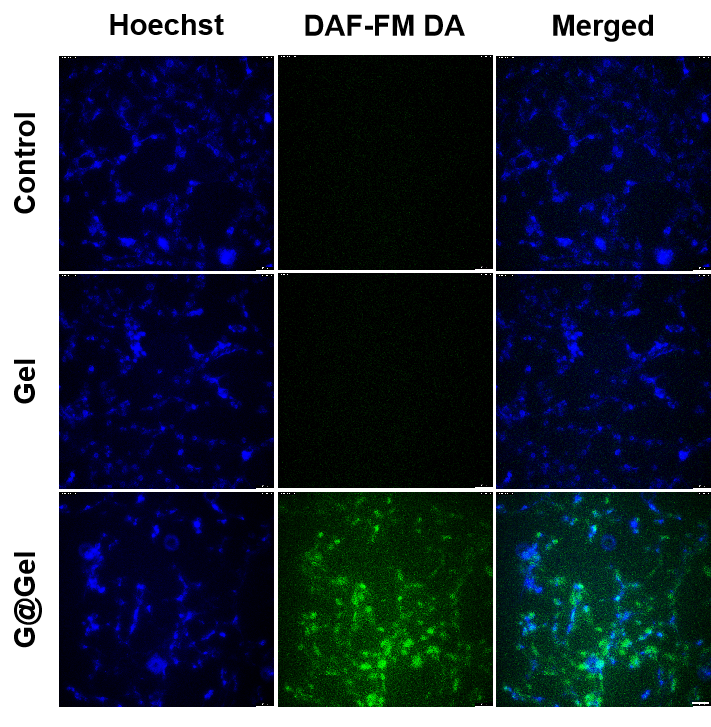


**Figure S18**. Uptake of NO by CAFs using DAF-FM DA to detective intracellular NO, Scale bar=50µm.

**Figure S19**. The differentiation of RAW264.7 to M1-type macrophages induced by G@Gel. RAW264.7 were stained with CD86 (Marker for M1-type macrophages). Data are shown as mean ± SD (n = 3).


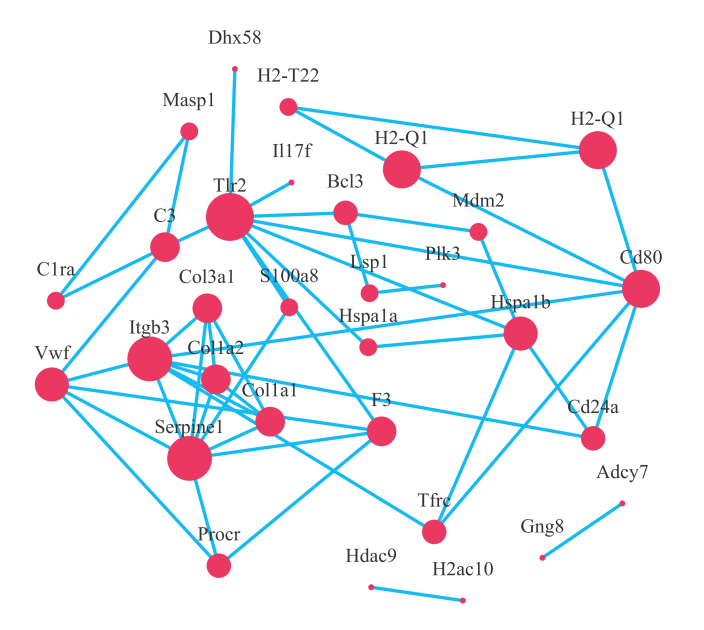


**Figure S20**. The analyses of functional interaction network of G@Gel regulated genes.

**Figure S21**. Temperature curve of the hydrogel irradiated with 808nm in 4T1 tumor-bearing mice. Data presented as mean ± SD (n = 3).


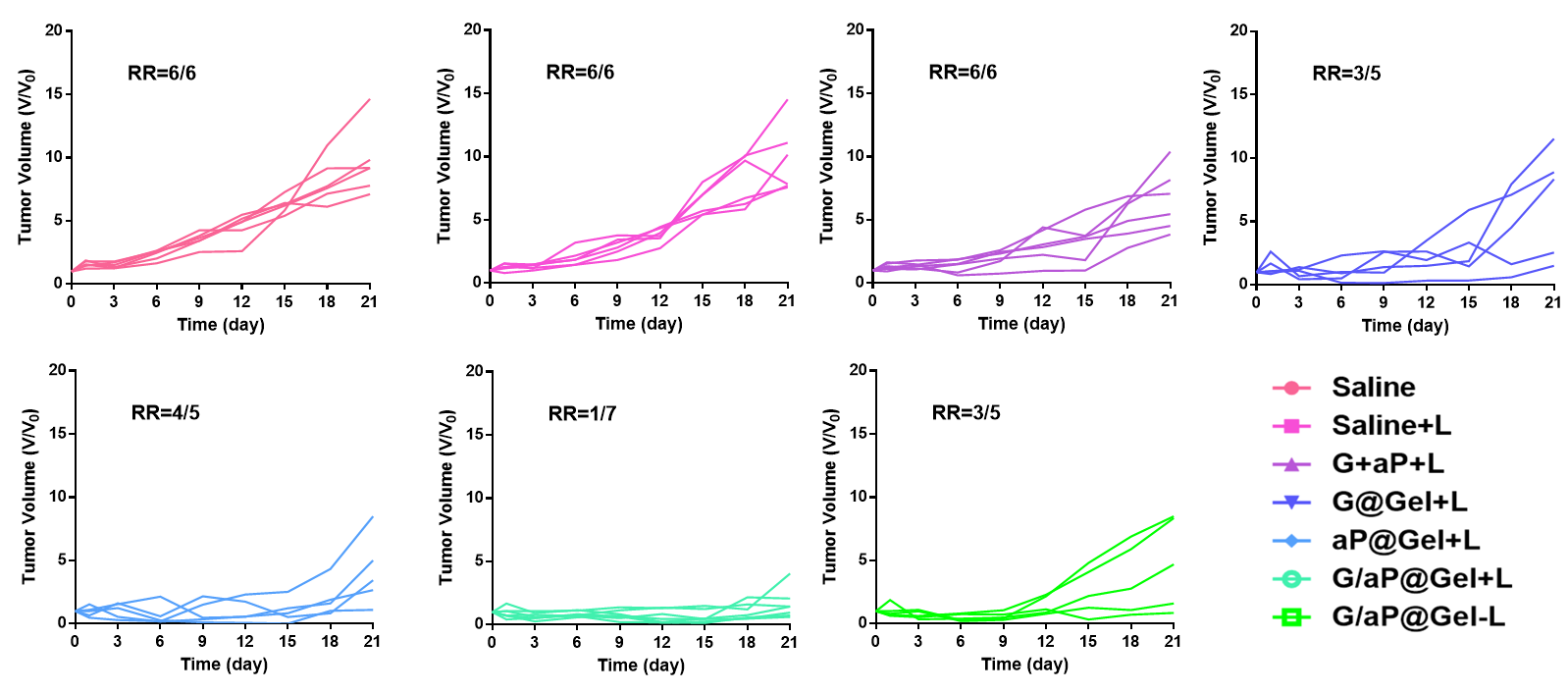


**Figure S22**. Growth curves of tumors in each treatments.


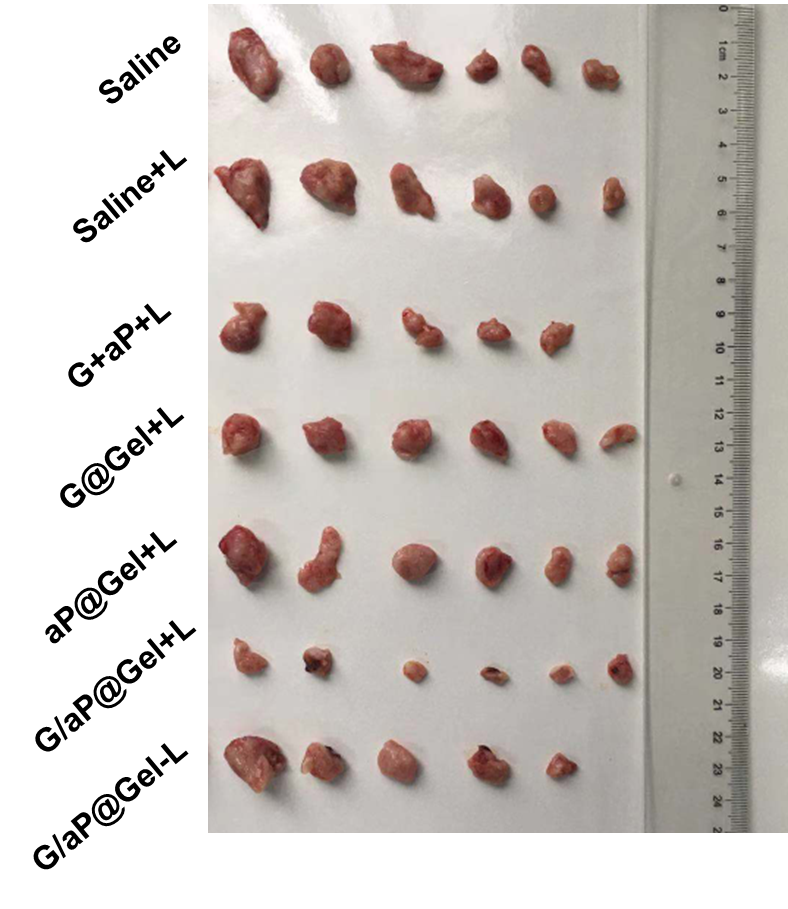


**Figure S23.** Photographs of tumors obtained from mice after 21 days treatment.

**
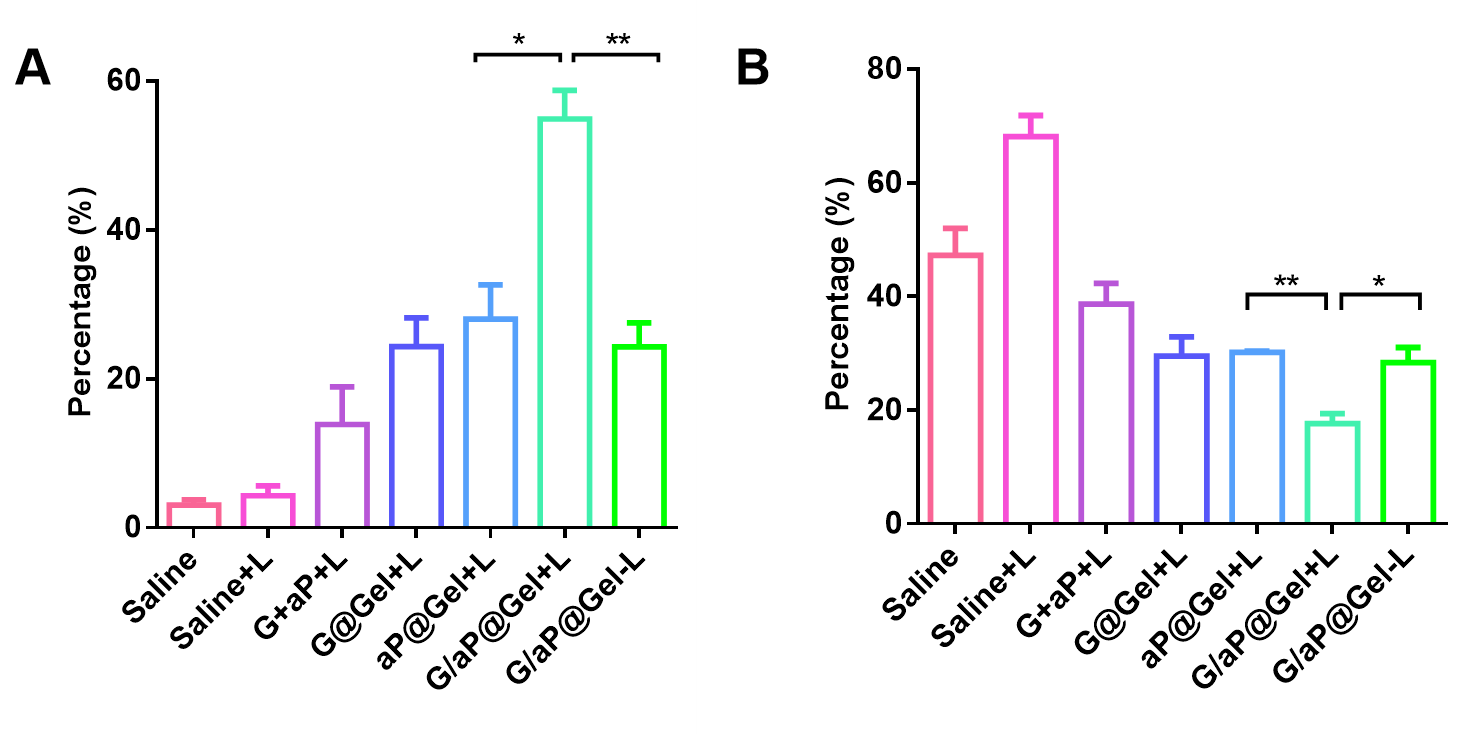
**

**Figure S24.** Relative (A) TUNEL and (B) Ki67 level in tumor immunofluorescence staining after various treatments. The comparison of two groups was followed by unpaired Student’s t-test (two-tailed). The level of significance was defined as *p < 0.05, **p < 0.01.


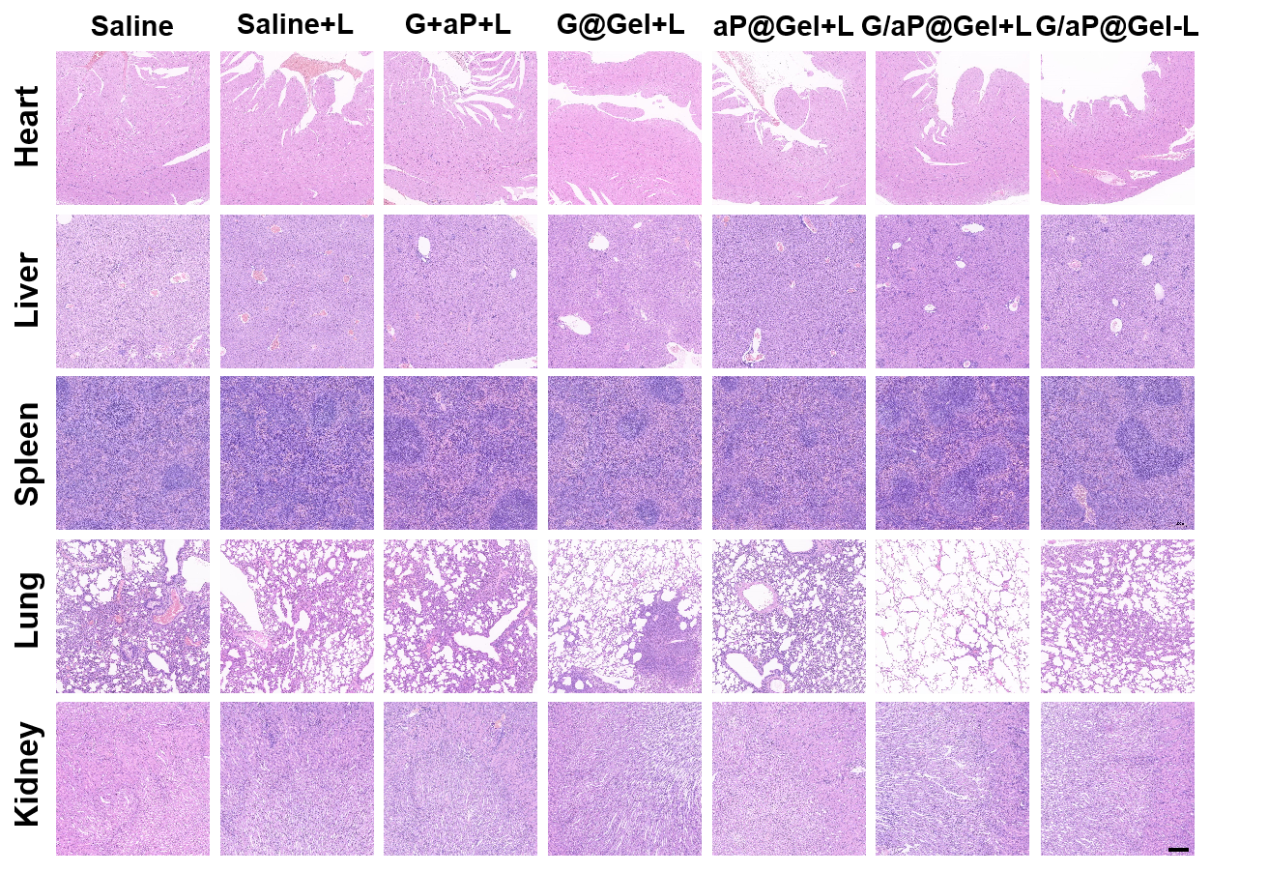


**Figure S25.** H&E staining of main organs on day 21 after various treatments, Scale bar=200µm.


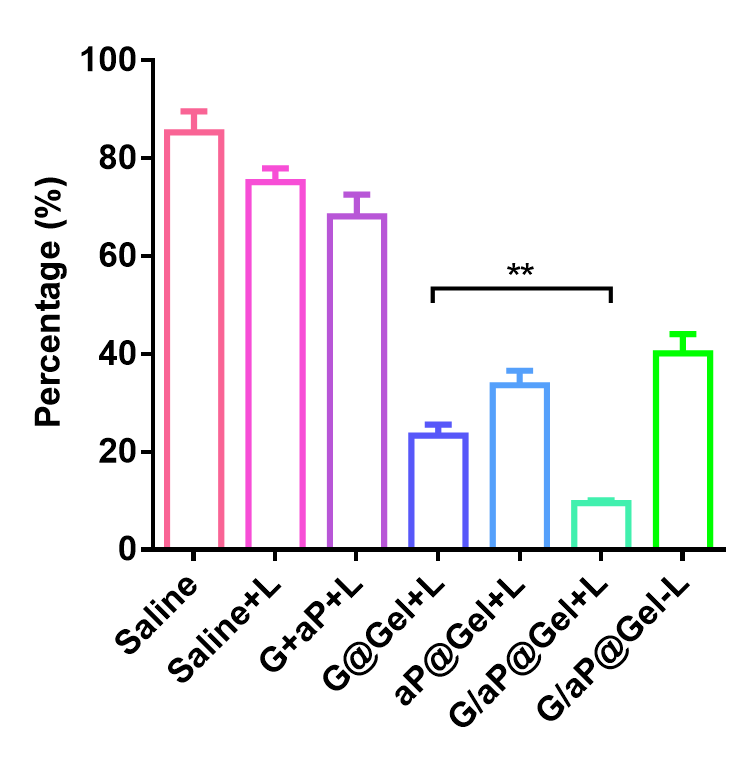


**Figure S26.**Relative α-SMA level in tumor immunofluorescence staining after various treatments. The comparison of two groups was followed by unpaired Student’s t-test (two-tailed). The level of significance was defined as *p < 0.05, **p < 0.01.


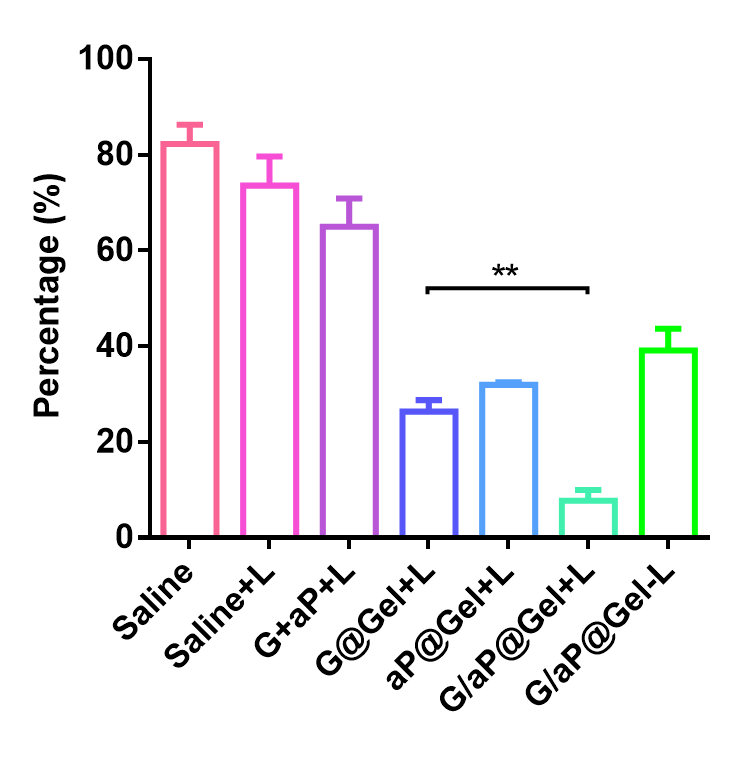


**Figure S27.**Relative Collagen Ⅰ level in tumor immunofluorescence staining after various treatments. The comparison of two groups was followed by unpaired Student’s t-test (two-tailed). The level of significance was defined as *p < 0.05, **p < 0.01.


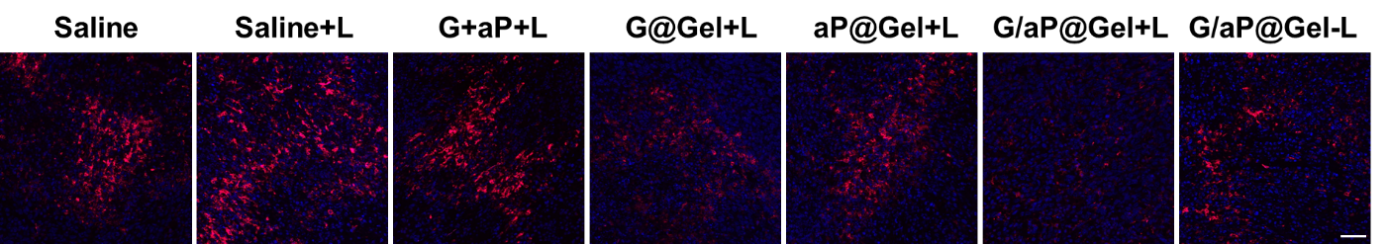


**Figure S28.** Images of immunofluorescence staining of M2 macrophages. DAPI, Blue; CD206, Red. Scale bar=50µm.


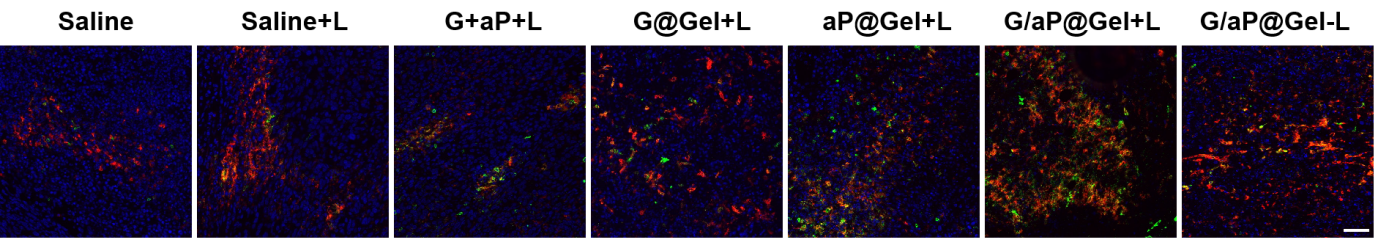


**Figure S29.** Images of immunofluorescence staining of M1 macrophages. DAPI, Blue; F4/80, Red; CD86, Green. Scale bar=50µm.


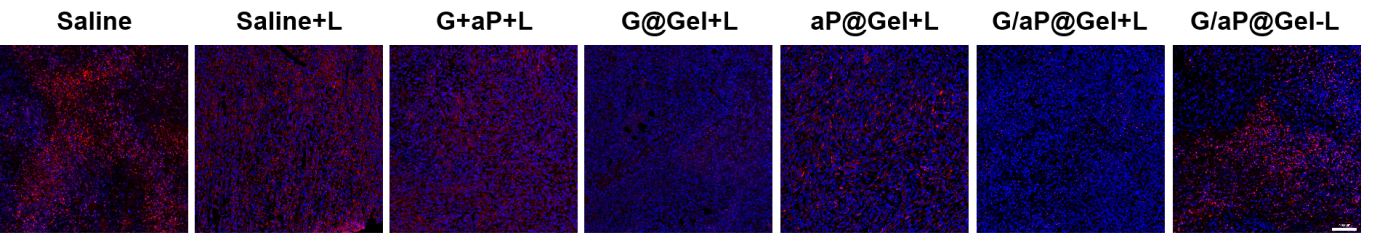


**Figure S30.** Images of immunofluorescence staining of TGF-β in different treatments, Scale bar=100µm.


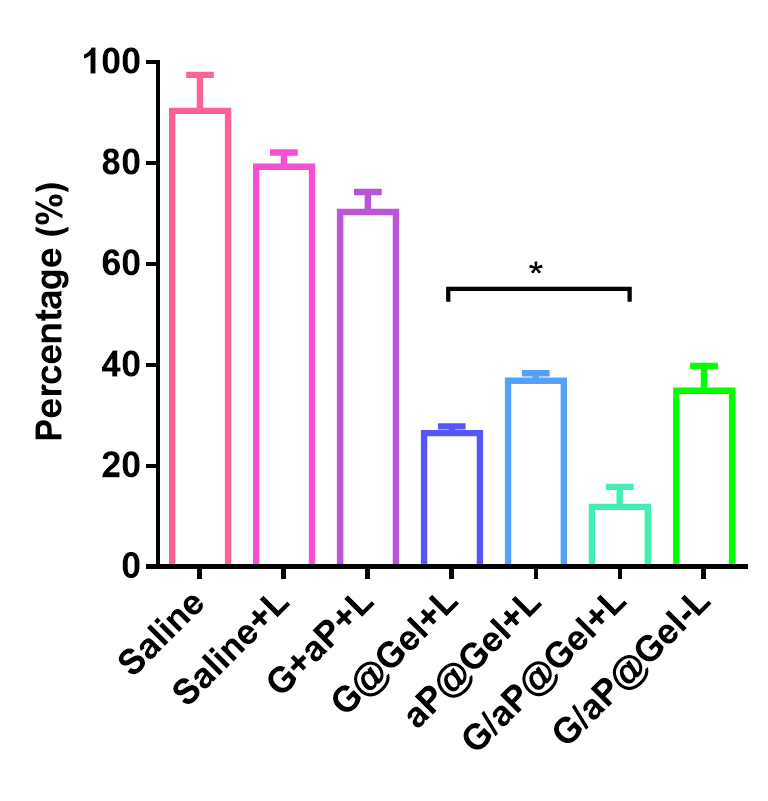


**Figure S31**.Relative TGF-β level in tumor immunofluorescence staining after various treatments. The comparison of two groups was followed by unpaired Student’s t-test (two-tailed). The level of significance was defined as *p < 0.05, **p < 0.01.

**Figure S32.** Relative HMGB1 level in tumor immunofluorescence staining after various treatments.


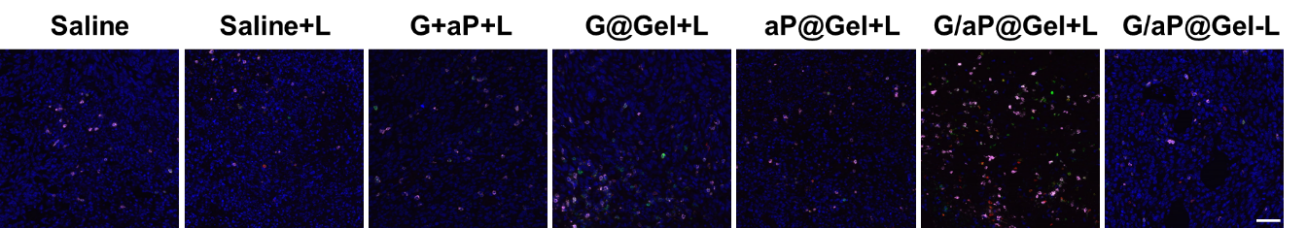


**Figure S33.** Images of immunofluorescence staining of T cells in tumor. DAPI, Blue; CD3, Green; CD4, Red; CD8, Pink. Scale bar=50µm.


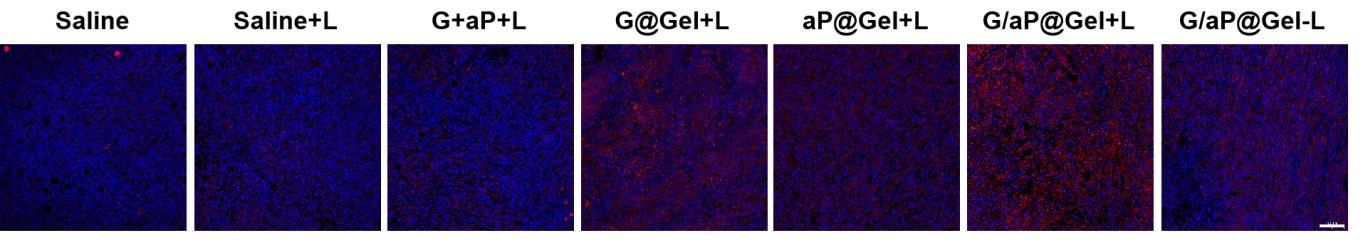


**Figure S34.** Images of immunofluorescence staining of TNF-α in tumor after various treatments, Scale bar=100µm.


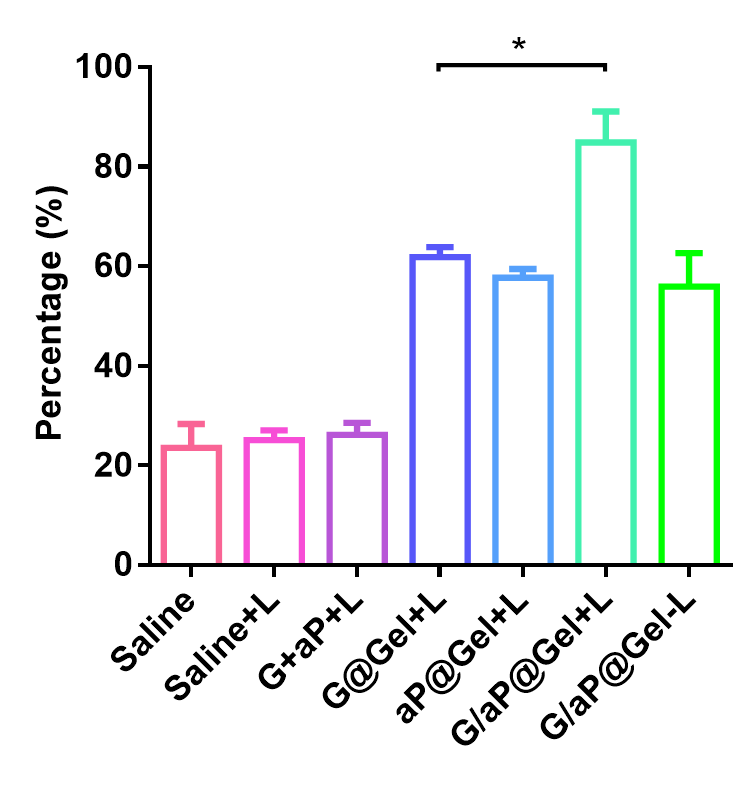


**Figure S35.** Relative TNF-α level in tumor fluorescence staining after various treatments. The comparison of two groups was followed by unpaired Student’s t-test (two-tailed). The level of significance was defined as *p < 0.05, **p < 0.01.


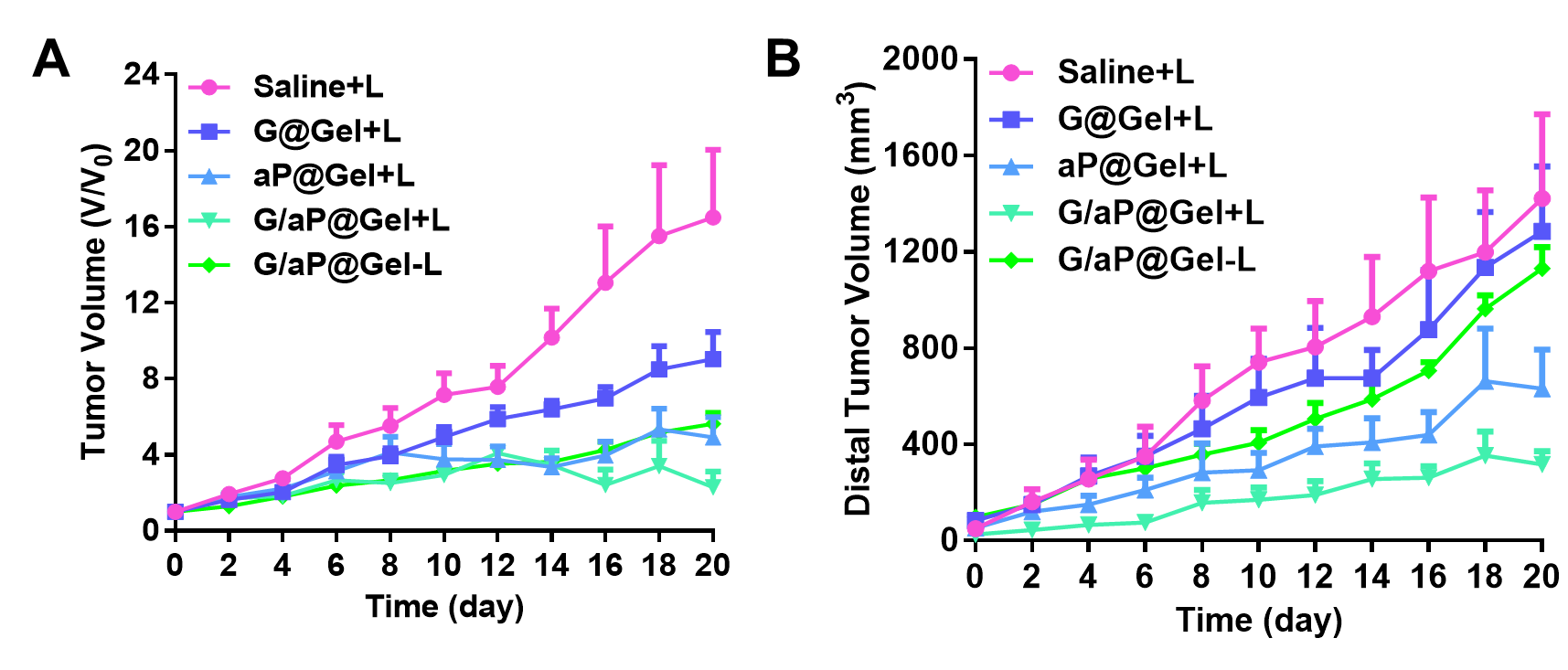


**Figure S36.** Growth curve of **(A)** primary tumor and **(B)** distant tumor after treatments.

**Figure S37.** Body weight of each group with different treatments.
